# A detailed insight in the high risks of hospitalizations in long-term childhood cancer survivors—A Dutch LATER linkage study

**DOI:** 10.1371/journal.pone.0232708

**Published:** 2020-05-19

**Authors:** Nina Streefkerk, Wim J. E. Tissing, Joke C. Korevaar, Eline van Dulmen-den Broeder, Dorine Bresters, Margriet van der Heiden-van der Loo, Marry M. van de Heuvel-Eibrink, Flora E. Van Leeuwen, Jacqueline Loonen, Helena H. J. van der Pal, Cecile M. Ronckers, A. Brigitta Versluys, Andrica C. H. de Vries, Elizabeth A. M. Feijen, Leontine C. M. Kremer, on behalf of the Dutch LATER Study Group

**Affiliations:** 1 Princess Máxima Center for Pediatric Oncology, Utrecht, The Netherlands; 2 Department Pediatric Oncology, Emma Children’s Hospital, Amsterdam UMC, University of Amsterdam, Amsterdam, The Netherlands; 3 Department of Pediatric Oncology/Hematology, Beatrix Children’s Hospital/University of Groningen/University Medical Center Groningen, Groningen, The Netherlands; 4 Netherlands Institute for Health Services Research, Utrecht, The Netherlands; 5 Department of Pediatric Oncology/Hematology, Amsterdam UMC, Vrije Universiteit Amsterdam, Amsterdam, The Netherlands; 6 Dutch Childhood Oncology Group, Utrecht, The Netherlands; 7 Department of Pediatric Oncology/Hematology, Sophia Children’s Hospital/Erasmus Medical Center, Rotterdam, The Netherlands; 8 Department of Epidemiology and Biostatistics, The Netherlands Cancer Institute, Amsterdam, The Netherlands; 9 Department of Hematology, Radboud University Medical Center, Nijmegen, The Netherlands; 10 Department of Pediatric Oncology and Hematology, Wilhelmina Children’s Hospital/University Medical Center Utrecht, Utrecht, The Netherlands; University of KwaZulu-Natal School of Social Sciences, SOUTH AFRICA

## Abstract

**Background:**

Insight in hospitalizations in long-term childhood cancer survivors (CCS) is useful to understand the impact of long-term morbidity. We aimed to investigate hospitalization rates and underlying types of diagnoses in CCS compared to matched controls, and to investigate the determinants.

**Methods:**

We linked 5,650 five-year CCS from the Dutch nationwide Dutch LATER cohort and 109,605 age- and sex-matched controls to the Dutch Hospital Discharge register, which contained detailed information on inpatient hospitalizations from 1995–2016. Relative hospitalization rates (RHRs) were calculated using a Poisson regression model. Adjusting for multiple hospitalizations per person via a Poisson model for generalized estimated equations, we investigated determinants for hospitalizations for all types of underlying diagnoses among CCS.

**Results:**

CCS were twice as likely to be hospitalized as reference persons (hospitalization rate 178 and 78 per 1,000 person-years respectively; RHR 2.0, 95% confidence interval (CI) 1.9–2.2). Although CCS had more hospitalizations for 17 types of underlying diagnoses, they were especially more likely to be hospitalized for endocrine conditions (RHR: 6.0, 95% CI 4.6–7.7), subsequent neoplasms (RHR: 5.6, 95% CI 4.6–6.7) and symptoms without underlying diagnoses (RHR: 5.2, 95% CI 4.6–5.8). For those types of underlying diagnoses, female sex and radiotherapy were determinants.

**Conclusion:**

This study provides new insights in the high risk of hospitalizations for many types of underlying diagnoses in CCS and treatment related determinants. CCS are especially at high risk for hospitalizations for endocrine conditions, subsequent neoplasms and symptoms without an underlying diagnosis. This new knowledge is important for survivorship care and to identify possible preventable hospitalizations among CCS.

## Introduction

Survival for childhood cancer has improved significantly over the past decades to about 80% nowadays. [1] Hence, the vast majority of childhood cancer patients will achieve long term survival and the number of long-term childhood cancer survivors will increase. Unfortunately, childhood cancer survivors (CCS) are at risk of developing long-term morbidity, such as subsequent malignancies, organ dysfunction, and endocrine disorders. [2–5] By the age of 50, a childhood cancer survivor has experienced an average of 4.7 severe health conditions, which is twice as many as in individuals that did not have cancer as a child. [5]

Insight in hospitalizations is useful to understand the impact of this long-term morbidity in CCS, because hospitalizations indicate severe morbidity that influence the patient’s daily life as well as healthcare costs. [6–9] Previous studies show that long-term CCS have a 1.5 to 3-fold higher rate of hospital admissions as compared to the general population. [10–17] Although several studies have established risk factors for specific long-term morbidity in CCS, it is unknown whether the same risk factors apply to the risk of hospitalizations for these type of conditions.

The aim of this study is to longitudinally evaluate the hospitalization rate and types of underlying conditions in a Dutch nationwide cohort of CCS, as compared to a matched reference population, and to identify treatment related risk factors for all types of underlying diagnoses among CCS.

## Methods

### Study population

We obtained our study population from the national Dutch Childhood Oncology Group—Long term Effects after Childhood Cancer (Dutch LATER) nationwide cohort, a collaborative effort of all Dutch pediatric oncology/hematology centers. This cohort includes 6,165 5-year CCS diagnosed with a malignancy according to the third edition of the International Classification of Childhood Cancer [18] before the age of 18 years, between 1/1/1963 and 12/31/2001, who were living in the Netherlands at the time of childhood cancer diagnosis and who were treated in one of the Dutch pediatric oncology/hematology centers. Details on cancer diagnosis and treatment schedules were retrospectively obtained from medical records using a standardized protocol. [19]

### Dutch Hospital Discharge register

The Dutch Hospital Discharge Register (Dutch acronym: LBZ) is maintained by Dutch Hospital Data and comprises data on hospital admission(s) of the Dutch population, from 1995 to 2016. [20] The LBZ contains data on date of admission and discharge, discharge diagnosis classified according to the International Classification of Diseases version 9 (ICD-9) and version 10 (ICD-10), and type of medical specialists involved. [21] Access to the LBZ is provided by Statistics Netherlands (Dutch acronym: CBS).

Until 2005, the coverage of the LBZ was > 96.7%. [22] After a slight decline in coverage, from 2013 onwards nearly all hospitalizations were registered in the LBZ, meaning that data on the total number of hospitalizations were nearly complete, but in in 5.5–21.4% of the cases, some of the data in individual hospitalization records were incomplete. In those records, information about one of the items for hospitalization was missing, for example discharge diagnosis, medical specialist at discharge or area where a person lived at time of hospitalization.

### Linkage procedure

A deterministic linkage method was performed as displayed in S1 Fig, using a unique identifier or a combination of sex, date of birth and postal code, if no identifier was available. CBS anonymized these identifying variables for all CCS into an anonymous unique record identification number (RIN) and removed all other identifying information. Because RIN was also the identifying variable in the LBZ, RIN was used to link LBZ data to clinical data. We removed CCS that had deceased before start of the LBZ from the dataset.

### Reference sample

A reference sample of the Dutch general population was obtained from the Municipal Personal Records Database (Dutch acronym: GBA). For each CCS, a maximum of 20 unique reference persons were selected with corresponding year of birth and sex. RINs were retrieved from all reference persons from the GBA and were used to retrieve their data from the LBZ. To determine start of follow-up, reference persons were assigned the date of diagnosis of their corresponding CCS.

### Ethical statement

Dutch law allows the use of Electronic Health Records for research purposes under certain conditions (Dutch Civil Law, Article 7: 458). According to this legislation, it is not necessary to obtain informed consent from patients or any form of approval or waiver from a medical ethics committee or institutional review board for this type of observational study that contains no directly identifiable data. This study was also reviewed by the Institutional Review Board of the Amsterdam UMC and was exempted from the need of ethical approval.

CBS provides access to the LBZ within a secured environment and ensures privacy protection by using RINs which prevents the possibility of exposing identity of specific individuals in the registration. According to Dutch LATER privacy regulations, data from CCS could be used after anonymizing, and data from CCS who explicitly refused the use of their data for linkage purposes were considered not eligible (n = 147). According to CBS confidentiality regulations, we do not present frequencies of less than 10.

### Definition of variables

Outcome of interest was the total number of hospitalizations per survivor from 1995 until 2016, defined as inpatient admissions of any duration. Hospitalizations for giving birth were excluded, as were outpatient clinic visits. Primary discharge diagnoses were categorized into organ systems according to the ICD-10 chapters. [23] If no discharge diagnosis was available from the LBZ, the ICD-10 chapter of the discharge diagnosis was assigned according to the type of medical specialist involved at discharge, or was categorized as missing when no information on type of medical specialist was available.

Time at risk started at five years after the primary cancer diagnosis or January 1, 1995, whichever came latest. Time at risk ended at date of death, date of emigration or December 31, 2015, whichever came first. Time during hospitalization was not counted as time at risk. CCS who had a recurrence of their primary childhood cancer beyond their five-year survival date were assumed to have an increased hospitalization rate due to treatment of their recurrence(s). Therefore, those CCS and their corresponding reference persons were censored at the date of recurrence of the childhood cancer, and were excluded if they were censored before start of follow-up (n = 28 CCS and n = 560 corresponding reference persons). Furthermore, 3,395 reference persons were excluded because they died or emigrated before 1-1-1995 or before start of follow-up and therefore did not contribute to time at risk.

Primary childhood cancer diagnosis was categorized into 9 subgroups of which a specification is available in S1 Table).

### Statistical analysis

Differences in characteristics between CCS and reference persons were assessed using Mann Whitney U tests when continuous and Pearson Chi squared tests when categorical. Hospitalization rates were calculated during the total time at risk for CCS and their matched reference persons per 1,000 person years (PY), both overall and per ICD-10 category. The absolute excess rate (AER) was calculated per 1,000 PY by subtracting the hospitalization rate from the reference population from the hospitalization rate from CCS. Using a Poisson regression model, Relative Hospitalization Rates (RHRs) were calculated adjusted for matched cases and controls and for multiple hospitalizations in one person.

Within the cohort of CCS, a multivariable Poisson regression model was built adjusting for multiple hospitalizations via Generalized Estimated Equations (GEE) to investigate determinants for hospitalizations. Separate models were executed for all underlying types of diagnoses except perinatal and congenital conditions, because we assumed that treatment of the primary childhood cancer did not influence these hospitalizations. In each model we included sex, age at diagnosis of primary cancer (categorical variable), follow-up time (continuous variable), 6 groups of chemotherapy, 9 locations of radiotherapy and surgery (specification in S1 Table). Two-sided p-values were reported and those of less than 0.05 were considered statistically significant. Analyses were performed using R (version 3.1.1, R Foundation) and SPSS (version 24, IBM SPSS Statistics).

## Results

### Study population

After excluding 208 CCS who died before 1-1-1995, and 28 CCS with a recurrence after five-year survival date that did not contribute to the time at risk (S1 Fig), a total of 5,650 CCS contributed 90,752 years at risk and 109,605 reference persons contributed 1,576,910 years at risk. The mean time from five year survival to end of follow-up was 17.9 years for CCS (interquartile range (IQR) 12.1–21.0) and 15.7 years for reference persons (IQR 10.1–21.0, Table 1).

**Table 1:** Patient, cancer and treatment characteristics of study population of five-year childhood cancer survivors and age and sex matched reference population.

	CCS study population (n = 5,650)		Reference population (n = 109,605)	
**Patient characteristics**				
*Sex*^1^—*n (%)*				
Male	3,152	55.8%	61,070	55.7%
Female	2,498	44.2%	48,535	44.3%
*Year of birth*^1^—*n (%)*				
<1970	589	10.4%	11,752	10.7%
1970–1985	2,748	48.6%	54,128	49.4%
>1985	2,131	37.7%	43,725	39.9%
**Tumor and treatment characteristics**				
*Age at diagnosis (in years)*^2^—*n (%)*				
0–4	2,557	45.3%	49,145	44.8%
5–9	1,531	27.1%	29,743	27.1%
10–14	1,203	21.3%	23,489	21.4%
15–17	359	6.4%	7228	6.6%
*Period of diagnosis*^2^—*n (%)*				
≤1974	343	6.1%	6,843	6.2%
1975–1984	1,353	23.9%	26,878	24.5%
1985–1994	2,055	36.4%	40,172	36.7%
1995–2002	1,899	33.6%	35,712	32.6%
*Primary childhood cancer*^3^—*n (%)*				
Leukemia	1,900	33.6%	*NA*	
Hodgkin lymphoma	383	6.8%	*NA*	
Non-Hodgkin lymphoma	543	9.6%	*NA*	
Central nervous system tumors	744	13.2%	*NA*	
Bone tumors	332	5.9%	*NA*	
Soft tissue sarcomas	406	7.2%	*NA*	
Renal tumors	567	10.0%	*NA*	
Neuroblastoma	303	5.4%	*NA*	
Other^4^	472	8.4%	*NA*	
*Treatment modality*^3^—*n (%)*				
Surgery only	568	10.1%	*NA*	
Chemotherapy ± surgery	2,839	50.2%	*NA*	
Radiotherapy ± surgery	432	7.6%	*NA*	
Chemotherapy + Radiotherapy ± surgery	1,765	31.2%	*NA*	
No therapy/therapy unknown	46	0.8%	*NA*	
*Chemotherapy*^5^—*n(%)*				
Anthracyclines	2,605	46.1%	*NA*	
Alkylating agents	2,878	50.9%	*NA*	
Platinum agents	736	13.0%	*NA*	
Vinca alkaloids	4,074	72.1%	*NA*	
Antimetabolites	2,618	46.3%	*NA*	
Epipodophyllotoxins	1,180	20.9%	*NA*	
*Radiotherapy—n (%)*				
Cranial radiotherapy^4^	1,193	21.1%	*NA*	
Radiotherapy to the neck^4^	218	3.9%	*NA*	
Radiotherapy to the spine^4^	355	6.3%	*NA*	
Radiotherapy to the thorax^5^	351	6.2%	*NA*	
Abdominopelvic radiotherapy^5^	420	7.4%	*NA*	
Radiotherapy to the upper extremities^6^	41	0.7%	*NA*	
Radiotherapy to the lower extremities^6^	73	1.3%	*NA*	
Total body irradiation^4^	200	3.5%	*NA*	
*Other therapies—n (%)*				
Hematopoietic stem cell transplantation	213	3.8%	*NA*	
**Follow-up**				
*Attained age at end of follow-up*, *in years—n (%)*				
< 20	752	13.3%	10,728	9.8%
20–30	1,899	33.6%	41,256	37.6%
30–40	1,777	31.5%	34,348	31.3%
40–50	990	17.5%	18,380	16.8%
> 50	232	4.1%	4,893	4.5%
*Time since 5-year survival to end of follow-up*, *in years—n (%)*				
5–9	846	15.0%	26,885	24.5%
10–14	1,364	24.1%	25,107	22.9%
14–19	1,061	18.8%	18,506	16.9%
20–25	2,379	42.1%	39,107	35.7%
*Years at risk (total number of years for each group)*	90,752		1,576,910	

Abbreviations: CCS: Childhood Cancer Survivors

^1^ Variables used for matching of CCS to the reference population

^2^ Age at diagnosis and treatment period were calculated for the reference population using the assigned date of diagnosis from their corresponding CCS

^3^ Variable options are mutually exclusive

^4^ Other tumors comprise (frequency tables are displayed in S1 Table):
Germ cell tumors, trophoblastic tumors, and neoplasms of gonads (*Gonadal carcinomas*, *Malignant gonadal germ cell tumors*, *Malignant extracranial and extragonadal germ cell tumors*, *Intracranial and intraspinal germ cell tumors*, *Other and unspecified malignant gonadal tumors*) Other malignant epithelial neoplasms and malignant melanomas (*Other and unspecified carcinomas*, *Skin carcinomas*, *Malignant melanomas*, *Nasopharyngeal carcinomas*, *Thyroid carcinomas*, *Adrenocortical carcinomas*) Langerhans cell histiocytosis Hepatic tumors (*Hepatic carcinomas*, *Hepatoblastoma*) Retinoblastoma Other and unspecified malignant neoplasms

^5^ For specification of chemotherapy variables, see S1 Table.^4^ Missing in 11 CCS.

^5^ Missing in 12 CCS.

^6^ Missing in 19 CCS.

### Hospitalization rates

**Fig 1:**
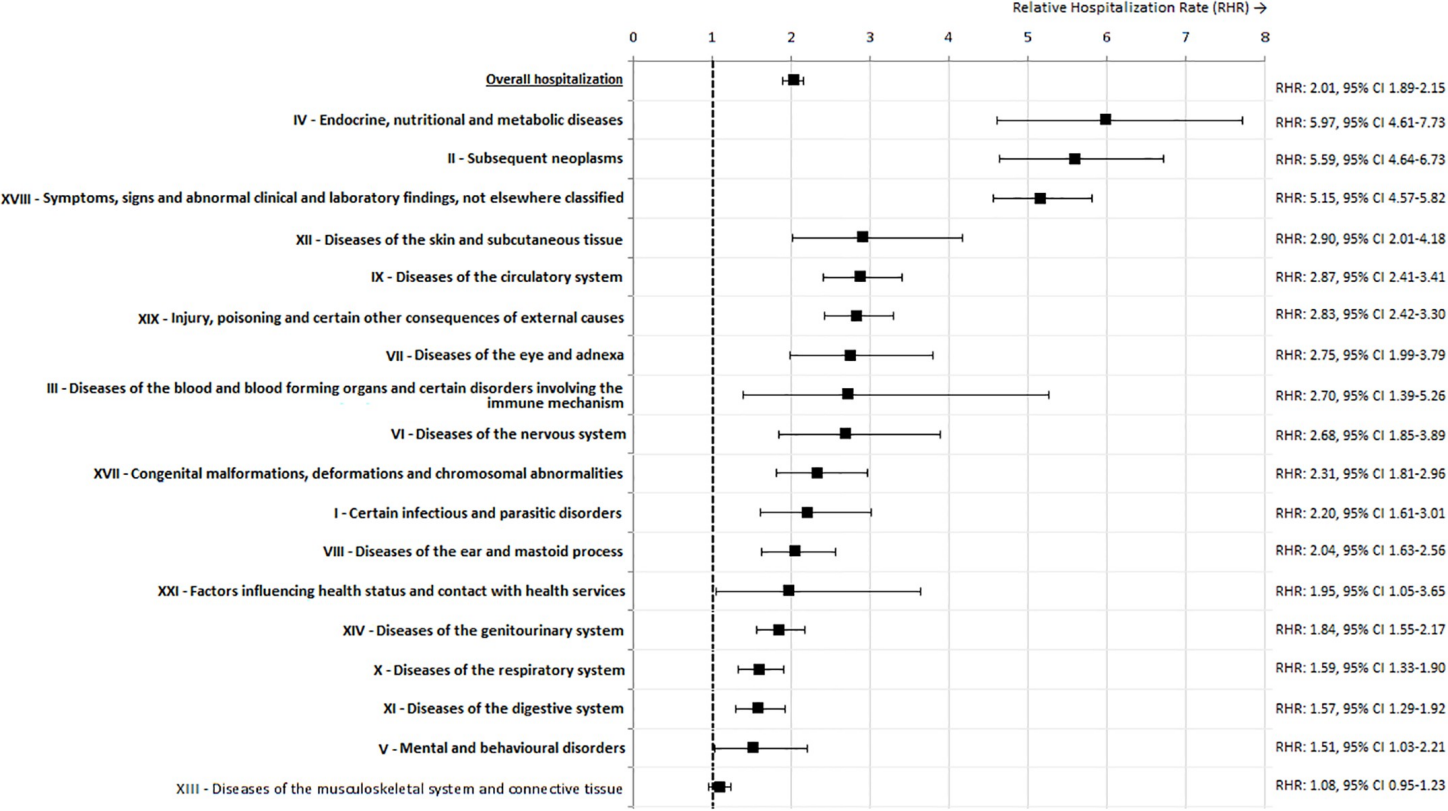
Relative hospitalization rates for five-year childhood cancer survivors as compared to the reference population, overall and for each type of hospitalization related health condition. Abbreviations: 95% CI: 95% confidence interval, RHR: relative hospitalization ratio.

**Fig 2:**
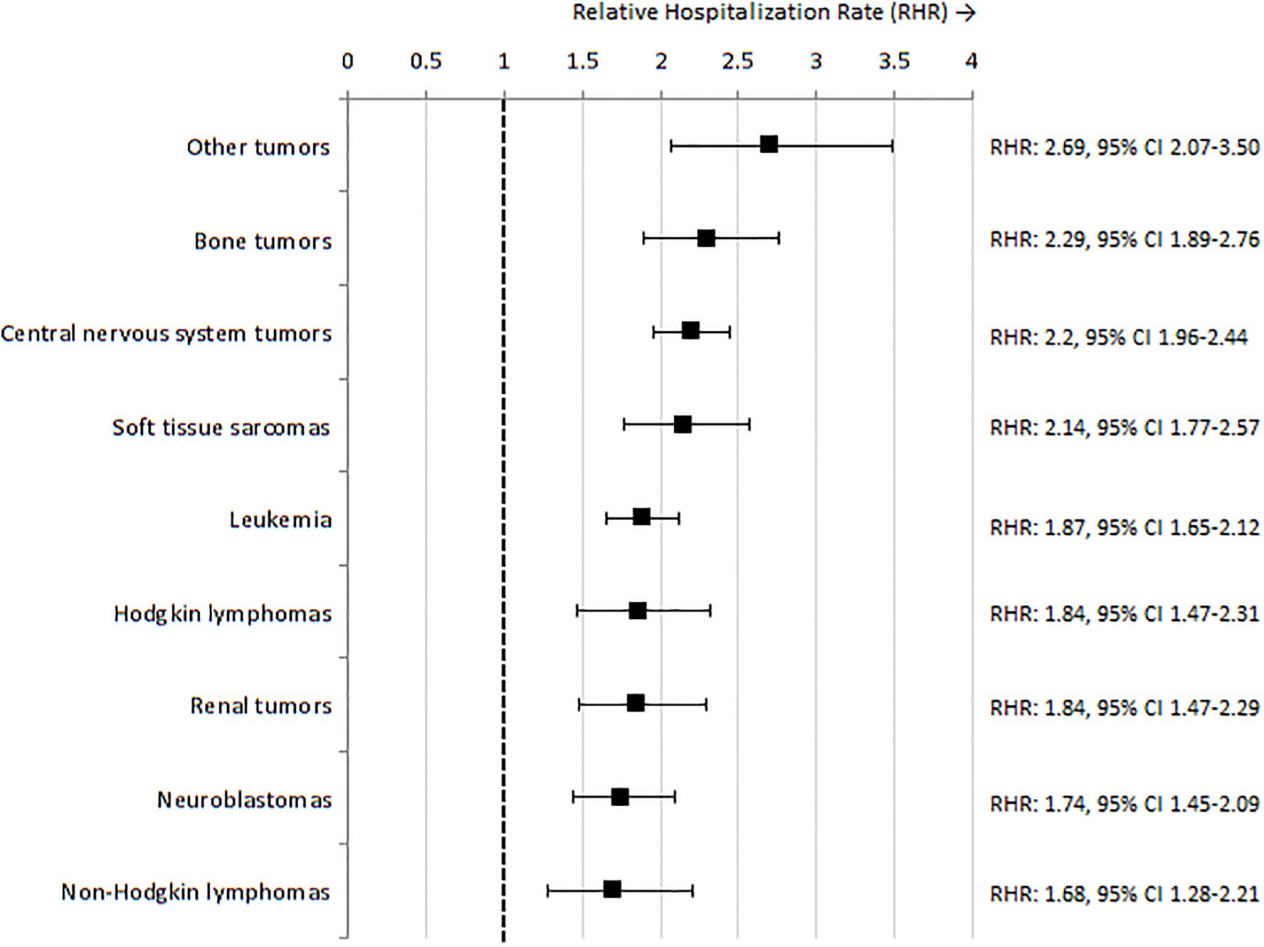
Relative hospitalization rates for five-year childhood cancer survivors as compared to the reference population, for by childhood cancer diagnosis.

A total of 16,141 hospitalizations were identified in CCS, resulting in an average rate of 177.9 hospitalizations per 1,000 PY (S2 Table). The average hospitalization rate in the reference population was 77.7 per 1,000 PY (S2 Table). CCS were hospitalized twice as often as the reference population (RHR: 2.01, 95% confidence interval (CI) 1.89–2.15, p<0.001, Fig 1, S2 Table). The AER was 100.18 per 1,000 PY in CCS, meaning that if 10 CCS were followed for one year, there was one extra hospitalization compared to the reference population. All CCS cancer diagnosis groups, and in particular bone tumors, central nervous system tumors and soft tissue sarcoma, were associated with a significantly increased hospitalization rate as compared to the reference population (Fig 2, S1 Table).

Other tumors comprise (frequency tables are displayed in S1 Table):

- Germ cell tumors, trophoblastic tumors, and neoplasms of gonads (*Gonadal carcinomas*, *Malignant gonadal germ cell tumors*, *Malignant extracranial and extragonadal germ cell tumors*, *Intracranial and intraspinal germ cell tumors*, *Other and unspecified malignant gonadal tumors*)
- Other malignant epithelial neoplasms and malignant melanomas (*Other and unspecified carcinomas*, *Skin carcinomas*, *Malignant melanomas*, *Nasopharyngeal carcinomas*, *Thyroid carcinomas*, *Adrenocortical carcinomas*)
- Langerhans cell histiocytosis
- Hepatic tumors (*Hepatic carcinomas*, *Hepatoblastoma*)
- Retinoblastoma
- Other and unspecified malignant neoplasms

Compared to the reference population, CCS had significantly higher hospitalization rates for 17 out of 18 types of discharge diagnoses (Fig 1, S1 Table). Relative to the reference population, CCS were most likely to be hospitalized for endocrine, nutritional and metabolic diseases (RHR: 5.97, 95% CI 4.61–7.73; Fig 1, S2 Table), including metabolic disorders, disorders of the adrenal gland, disorders of the thyroid gland, and other endocrine disorders (S3 Table). CCS were second most likely to be hospitalized for subsequent neoplasms (RHR: 5.59, 95% CI 4.64–6.73; Fig 1, S2 Table), among which were subsequent malignant neoplasms, benign neoplasms, carcinoma in situ and neoplasms of uncertain behavior (S3 Table). Symptoms, signs and abnormal clinical findings not elsewhere classified, i.e. symptoms without an underlying diagnosis, led to over 5 times as many hospitalizations in CCS as in the reference population (RHR: 5.15, 95% CI 4.57–5.82, Fig 1, S2 Table) and the AER was 24.45, meaning that if 40 CCS are followed for one year, there was one extra hospitalization compared to the reference population. Diseases of the skin and subcutaneous tissue and diseases of the circulatory system also had high RHRs (RHR: 2.90, 95% CI 2.01–4.18 and RHR: 2.87, 95% CI 2.41–3.41 respectively, Fig 1, S2 Table). The three most prevalent conditions of the circulatory system for which CCS were hospitalized, according to S3 Table, were classified as other forms of heart disease (including acute rheumatic fever, chronic rheumatic heart disease; n = 110), Other diseases of veins and lymphatics, and other diseases of circulatory system (n = 79), Cerebrovascular disease (n = 67).

Significantly more CCS than reference persons experienced at least one hospitalization without an underlying diagnosis (n = 1,188, 21.0% and n = 5,895, 5.4% respectively, p = 0.001; S5 Table) and 458 CCS (8.1%) experienced two or more. All hospitalizations for symptoms without an underlying diagnosis were classified into respective organ systems, or were labeled as “other or unknown” if the discharge diagnosis was unclear. The latter occurred significantly more often for hospitalizations among CCS than among reference persons (n = 1,863/2,722 (68.4%) hospitalizations and n = 2,058/8,471 (24.3%) hospitalizations respectively, p<0.001, S6 Table).

### Determinants for higher hospitalization rates

Table 2 displays the outcomes of the multivariable model investigating determinants for hospitalizations for the five types of underlying diagnoses with the highest RHRs in CCS (Fig 1). Determinants for endocrine, metabolic and nutritional disorders were female sex (RHR 1.50, 95% CI 1.03–2.17), cranial radiotherapy (RHR: 2.69, 95% CI 1.57–4.63) and abdominopelvic radiotherapy (RHR: 2.51, 95% CI 1.53–4.13). For hospitalizations for subsequent neoplasms, determinants were female sex (RHR: 1.80, 95% CI 1.30–2.50), cranial radiotherapy (RHR: 1.85, 95% CI 1.76–2.94), abdominopelvic radiotherapy (RHR: 1.72, 95% CI 1.13–2.63), radiotherapy to the lower extremities (RHR: 2.04, 95% CI 1.10–3.80) and treatment with epipodophyllotoxins (RHR 1.73, 95% CI 1.06–2.84;Table 2). For hospitalizations for diseases of the skin and subcutaneous tissue, no treatment related determinants were identified. Cranial radiotherapy (RHR: 1.74, 95% 1.16–2.59), radiotherapy to the thorax (RHR: 2.94, 95% CI 1.80–4.82) and lower extremities (RHR: 3.79, 95% CI 1.85–7.79) were determinants for hospitalizations because of cardiovascular diseases (including ischemic heart disease, cardiovascular disease, hypertension, and other circulatory disorders), as were treatment with anthracyclines (RHR: 1.56, 95% CI 1.11–2.19) and alkylating agents (RHR: 1.51, 95% CI 1.05–2.16). Results of multivariable models for all other types of underlying diagnoses are displayed in S3 Table.

**Table 2:** Multivariable risk factor analyses for the effect of treatment related risk factors on the number of hospitalizations among childhood cancer survivors. For each category of hospitalization related health conditions, a separate Poisson regression model was performed to evaluate treatment related risk factors (S3 Table). This table displays the outcomes of the risk factor analyses for four of the types of hospitalization related health conditions with the highest relative hospitalization rates in CCS as compared to the reference population. Risk factor analyses were conducted among CCS in which treatment details were known (n = 5,607).

	IV—Endocrine, nutritional and metabolic diseases				II—Subsequent neoplasms				XVIII—Symptoms, signs and abnormal clinical and laboratory findings, not elsewhere classified				XII—Diseases of the skin and subcutaneous tissue				IX—Diseases of the circulatory system			
	n/n with event	RHR	95%CI	p-value	n/n with event	RHR	95%CI	p-value	n/n with event	RHR	95%CI	p-value	n/n with event	RHR	95%CI	p-value	n/n with event	RHR	95%CI	p-value
**Sex**^**1**^																				
Male	3125/163	Ref			3125/285	Ref			3125/592	Ref			3125/101	Ref			3125/163	Ref		
Female	2482/199	**1.499**	**1.034–2.172**	**0.033**	2482/349	**1.804**	**1.302–2.501**	**0.000**	2482/591	**1.294**	**1.042–1.606**	**0.020**	2482/116	1.012	0.571–1.795	0.967	2482/130	0.921	0.664–1.278	0.623
**Age at diagnosis,years**^1^																				
0–4	2543/166	Ref			2543/253	Ref			2543/551	Ref			2543/99	Ref			2543/94	Ref		
5–9	1519/122	1.312	0.784–2.196	0.301	1519/167	0.896	0.633–1.268	0.535	1519/322	0.836	0.631–1.107	0.211	1519/59	0.668	0.369–1.208	0.182	1519/81	1.055	0.598–2.031	0.805
10–14	1193/56	0.770	0.395–1.499	0.441	1193/162	1.318	0.885–1.963	0.174	1193/234	**0.633**	**0.493–0.814**	**<0.001**	1193/44	0.572	0.319–1.023	0.060	1193/87	1.235	0.790–1.933	0.355
15–17	352/18	0.661	0.301–1.452	0.303	352/52	1.314	0.775–2.227	0.311	352/76	**0.543**	**0.391–0.753**	**<0.001**	352/15	1.225	0.336–4.465	0.758	352/31	1.102	0.690–1.613	0.756
**Follow-up time**		**1.066**	**1.015–1.119**	**0.010**		0.981	0.960–1.003	0.084		1.011	0.992–1.031	0.236		**1.120**	**1.072–1.171**	**<0.001**		**1.106**	**1.068–1.146**	**<0.001**
**Surgery**	3797/258	1.550	0.701–3.427	0.279	3797/448	1.149	0.716–1.845	0.565	3797/931	**2.156**	**1.787–3.544**	**<0.001**	3797/159	1.431	0.483–4.239	0.517	3797/221	0.779	0.472–1.286	0.329
**Radiotherapy**																				
Cranial RT	1193/156	**2.694**	**1.567–4.632**	**<0.001**	1193/237	**1.847**	**1.163–2.935**	**0.009**	1193/321	**2.043**	**1.575–2.649**	**<0.001**	1193/51	0.551	0.309–0.985	0.044	1193/95	**1.737**	**1.164–2.592**	**0.007**
Spinal RT	355/58	1.328	0.596–2.959	0.488	355/74	1.538	0.684–3.459	0.297	355/106	1.007	0.680–1.492	0.971	355/20	1.423	0.671–3.018	0.358	355/29	0.891	0.513–1.546	0.681
Total body irradiat.	200/27	2.646	0.875–8.005	0.085	200/44	1.956	0.907–4.216	0.087	200/53	1.439	0.819–2.527	0.205	200/<10	**0.115**	**0.025–0.529**	**0.005**	200/11	2.128	0.727–6.222	0.168
RT thorax	351/26	0.635	0.353–1.142	0.129	351/69	0.914	0.382–2.188	0.840	351/86	**0.692**	**0.494–0.970**	**0.033**	351/17	1.136	0.529–2.440	0.743	351/56	**2.944**	**1.798–4.822**	**<0.001**
Abdominalpelvic RT	420/49	**2.514**	**1.532–4.128**	**<0.001**	420/76	**1.723**	**1.130–2.628**	**0.012**	420/101	**1.792**	**1.077–2.983**	**0.025**	420/18	0.465	0.194–1.113	0.085	420/40	1.029	0.620–1.705	0.913
Neck RT	218/15	0.980	0.486–1.975	0.956	218/38	2.245	0.641–7.863	0.206	218/59	**1.388**	**1.010–1.908**	**0.043**	218/<10	0.365	0.130–1.026	0.056	218/29	1.544	0.833–2.865	0.168
RT Upper extremities	41/<10	0.228	0.033–1.598	0.137	41/11	1.663	0.779–3.462	0.174	41/<10	0.420	0.174–1.011	0.053	41/<10	0.787	0.185–3.340	0.745	41/<10	0.845	0.353–2.023	0.705
RT Lower extremities	73/<10	1.319	0.441–3.940	0.620	73/19	**2.044**	**1.100–3.798**	**0.024**	73/15	0.816	0.412–1.614	0.559	73/<10	1.207	0.380–3.839	0.750	73/14	**3.793**	**1.848–7.787**	**<0.001**
**Chemotherapy**																				
Anthracyclines	2605/362	0.856	0.468–1.564	0.613	2605/283	1.161	0.815–1.655	0.409	2605/529	0.800	0.544–1.178	0.259	2605/102	1.365	0.546–3.414	0.506	2605/293	**1.558**	**1.108–2.192**	**0.011**
Alkylating agents	2878/181	1.125	0.683–1.856	0.643	2878/318	0.916	0.679–1.237	0.569	2878/612	1.106	0.813–1.503	0.521	2878/106	0.525	0.241–1.141	0.104	2878/158	**1.507**	**1.051–2.161**	**0.026**
Platinum	736/56	1.314	0.441–3.913	0.624	736/115	0.850	0.446–1.621	0.623	736/194	0.921	0.582–1.458	0.726	736/31	1.463	0.682–3.138	0.329	736/36	1.202	0.674–2.144	0.533
Vinca alkaloids	4074/249	0.800	0.463–1.383	0.424	4074/429	0.805	0.573–1.132	0.212	4074/804	0.828	0.665–1.031	0.091	4074/145	1.559	0.788–3.085	0.203	4074/190	**0.615**	**0.407–0.931**	**0.022**
Antimetabolites	2618/154	1.191	0.645–2.199	0.577	2618/271	1.035	0.641–1.671	0.890	2618/490	1.077	0.772–1.503	0.663	2618/91	0.942	0.319–2.777	0.913	2618/98	**0.471**	**0.274–0.810**	**0.006**
Epipodophyllotoxins	1180/362	1.756	0.634–4.859	0.278	1180/156	**1.732**	**1.057–2.837**	**0.029**	1180/309	**2.188**	**1.420–3.372**	**<0.001**	1180/39	2.499	0.982–6.358	0.055	1180/47	0.816	0.483–1.380	0.449

Abbreviations: 95% CI: 95% confidence interval, CCS: Childhood Cancer Survivors, POP: reference population, RHR: relative Hospitalization Ratio, RT: radiotherapy

^1^ Groups are mutually exclusive

For hospitalizations because of symptoms without an underlying diagnosis, treatment related risk factors are displayed in Table 2. An additional Poisson regression model, also including primary cancer diagnosis showed that the risk of hospitalizations for symptoms without underlying diagnosis was significantly increased for central nervous system tumor survivors (RHR: 2.95, 95% CI 2.10–4.13), survivors of soft tissue sarcoma (RHR: 1.69, 95% CI 1.11–3.45) and survivors of other tumors (RHR: 2.23, 95% CI 1.51–3.30; S8 Table).

## Discussion

This large study, in which a Dutch nationwide cohort of 5,650 long-term CCS and 109,605 matched reference persons were linked to the Dutch Hospital Discharge register, provides unique detailed insight in the increased risk and determinants of many types of hospitalizations. An important finding is the high risk of CCS for hospitalizations for symptoms without an underlying diagnosis.

For this study, we were able to link 92.1% of the nationwide Dutch LATER cohort to an administrative registry, creating an dataset without selection bias, containing detailed information on CCS’s characteristics and their hospitalizations. This large dataset provided sufficient statistical power for detailed investigation of determinants for 17 types of underlying diagnoses among CCS and for systematical assessment of hospitalizations for symptoms without an underlying diagnosis. By selecting ≤20 matched reference persons per CCS, we were able to relate CCS’s hospitalization rates to the general population. We adjusted for multiple hospitalizations within one person, using Generalized Estimated Equations (GEE).

We found that survivors experience significantly increased hospitalization rates, especially for endocrine conditions, and subsequent neoplasms. These results confirm previous studies, in which second neoplasms [13–16, 24–26], endocrine conditions [14, 15, 24, 26], conditions of the blood and blood forming organs [13, 16, 24, 25] and cardiovascular conditions [14, 15, 26] were shown to lead to hospitalizations in CCS. We extended these previous hospitalization investigations by showing that CCS experienced higher hospitalization rates for nearly all underlying types of conditions, and by investigating the treatment related determinants for each type of diagnosis leading to hospitalization in CCS in detail. For hospitalizations for endocrine, nutritional and metabolic conditions, cranial and abdominopelvic radiotherapy were treatment related determinants. We hypothesize this can be explained by the high prevalence of adrenal conditions, thyroid conditions and other endocrine deficiencies, which can primary (caused by damage to specific organs) or central (caused by damage to the hypothalamic/pituitary region). These results add important insight to previous literature, in which only radiotherapy to the head/neck was found to be a determinant for hospitalizations for endocrine conditions. [26] For hospitalizations because of subsequent neoplasms we identified cranial, abdominopelvic, and lower extremity radiotherapy and treatment with epipodophyllotoxins (which include teniposide and etoposide) as determinants. Although epipodophyllotoxins have well-established leukemogenic properties [27, 28]; they were not associated with risk of subsequent malignant neoplasms in previous analyses in our cohort. [19] We furthermore found that CCS were nearly three times as likely as the reference population to be hospitalized because of cardiovascular conditions in comparison to our previous study, we confirmed that treatment with radiotherapy to the thorax was a determinant, but we identified radiotherapy to the head as a new determinant. [26] Although radiotherapy to the head was not previously identified as a risk factor for hospitalization for cardiovascular conditions, previous literature showed that CCS treated with radiotherapy to the head have an increased risk of stroke. [29–32] Also, it is suggested that radiotherapy to the head can result in a low growth hormone level [33], which is likely to contribute to the development of metabolic syndrome [34] and, by being a modulator of myocardial structure and function [35], is associated with a higher cardiovascular risk for subgroups of CCS, for example ALL survivors treated with cranial radiotherapy [36] Moreover, we found that radiotherapy to the lower extremities also was associated with a higher risk of hospitalizations for cardiovascular conditions, which was not shown before in literature. We hypothesize this is due to venous diseases, comprising the second most prevalent circulatory condition among CCS. The new insights in determinants for all causes of hospitalizations in CCS as described in this study provide important new leads for in-depth investigation of determinants for hospitalizations for specific causes. Until now this knowledge was lacking.

Another important finding in this study is the high risk of hospitalizations for symptoms without an underlying diagnosis in CCS compared to the reference population, especially in survivors of central nervous system tumors. We looked in detail into the types of symptoms for these hospitalizations (S6 Table), and we found that hospitalizations were more often registered as for ‘other symptoms’ or ‘symptoms unknown’ in CCS than in the reference population, implicating that in CCS the underlying cause for hospitalization is often unclear. CCS might experience clinical symptoms that are unusual for their age range. This, in combination with their medical history of cancer, might cause clinicians to be more likely to hospitalize CCS for diagnostic evaluation when there are symptoms without a clear diagnosis. Hence, CCS’s medical background can introduce more precautious clinical decision-making. Furthermore, CCS might also have a lower threshold for consulting a physician than individuals who did not experience cancer as a child. Further research should determine whether a part of these hospitalizations might be preventable.

A limitation of using data from the LBZ, is that the longitudinal outcome data was available from 1995 onwards, implicating that data on hospitalizations for the older individuals might have been missing. This combined with the slight decline in coverage of the LBZ between 2005 and 2013, might have led to an underestimation of the hospitalization rates in both CCS and reference persons. We have no data that suggests that decline in coverage is higher for certain types of conditions leading tot hospitalizations. Moreover, there is no reason to assume that this decline is different for CCS, relative to other groups in the population and therefore, risk estimates are valid. Also, data on hospitalizations was available from 1995 onwards, which implicates that for CCS diagnosed in the earlier decades and their corresponding reference persons, data on hospitalizations in their early follow-up years might be missing. This could have led to an underestimation of the hospitalization rate in those groups. Because we matched reference persons for each CCS based on date of diagnosis and age of diagnosis, we expect the RHR estimates to be valid. Furthermore, since we present the results of many tests of statistical significance, we caution against over interpretation of our findings, especially those based on P values exceeding 0.001.

The detailed new knowledge on hospitalizations, causes and determinants in CCS as presented in this study will support the development of strategies for prevention of excess hospitalizations among CCS. This study also provided unique new insights in hospitalizations for symptoms without an underlying diagnosis and its determinants, thereby providing knowledge on possible preventable hospitalizations among CCS.

## Supporting information

S1 Fig Flow diagram linking individuals in the Dutch LATER cohort and a selected matched reference population to the Dutch Hospital Discharge register (LBZ). Abbreviations: CBS: Statistics Netherlands, CCS: Childhood Cancer Survivors, Dutch LATER: Dutch Childhood Oncology Group—Long term Effects after Childhood Cancer, LBZ: Dutch Hospital Discharge register, RIN: record identification number (assigned by CBS). ^1^: Statistics Netherlands (CBS) pseudonimized all identifying variables for all CCS into an anonymous unique record identification number (RIN) and hereafter removed all identifying information from the dataset. Because the RIN was also the identifying variable in the Dutch Hospital Discharge register (LBZ), the RIN was used to link LBZ data to clinical data.
(TIF)

S1 Table Definition of variables. (DOCX)

S2 Table Hospitalizations in five year childhood cancer survivors and in the reference population, relative hospitalization risks and absolute access risks for overall hospitalizations and for hospitalization associated health condition type. Abbreviations: 95% CI: 95% confidence interval, AER: Absolute Access Risk, CCS: Childhood Cancer Survivors, POP: reference population, PY: Person-Year, RHR: relative Hospitalization Ratio. Relative Hospitalization Ratios were adjusted for matched cases and controls, and for multiple hospitalizations.
(DOCX)

S3 Table Specification of underlying types of health conditions. (DOCX)

S4 Table Hospitalizations in five-year childhood cancer survivors and in the reference population, relative hospitalization risks and absolute access risks, per childhood cancer diagnosis. ^1^: Hospitalization rate in reference population: 77.68/1,000 PY Abbreviations: 95% CI: 95% confidence interval, AER: Absolute Access Risk, CCS: Childhood Cancer Survivors, POP: reference population, PY: Person-Year, RHR: relative Hospitalization Ratio. Relative Hospitalization Ratios were adjusted for matched cases and controls, and for multiple hospitalizations. *Other tumors comprise (for frequency table, see S1 Table):
Germ cell tumors, trophoblastic tumors, and neoplasms of gonads (Gonadal carcinomas, Malignant gonadal germ cell tumors, Malignant extra cranial and extra gonadal germ cell tumors, Intracranial and intraspinal germ cell tumors, Other and unspecified malignant gonadal tumors)Other malignant epithelial neoplasms and malignant melanomas (Other and unspecified carcinomas, Skin carcinomas, Malignant melanomas, Nasopharyngeal carcinomas, Thyroid carcinomas, Adrenocortical carcinomas)Langerhans cell histiocytosisHepatic tumors (Hepatic carcinomas, Hepatoblastoma)RetinoblastomaOther and unspecified malignant neoplasms
(DOCX)

S5 Table Frequency table of the total number of hospitalizations per person for hospitalizations because of symptoms without an underlying diagnosis among childhood cancer survivors and among the reference population. Chi square: p<0.001 Abbreviations: CCS: childhood cancer survivors, POP: reference population.
(DOCX)

S6 Table Summary of types of discharge diagnosis for all hospital admissions because of symptoms, signs and abnormal clinical findings among childhood cancer survivors and among the reference population. Chi square: p<0.001 Abbreviations: CCS: childhood cancer survivors. Frequencies of all hospitalizations for each specific ICD-10 code were listed, specific ICD-10 codes were grouped into categories of health conditions and presented in this table. This table sums the total number of hospitalizations and not the number of individual; one individual can contribute multiple hospitalizations.
(DOCX)

S7 Table Multivariable risk factor analyses for the effect of treatment related risk factors on the number of hospitalizations among childhood cancer survivors. For each category of hospitalization related health conditions, a separate Poisson regression model was performed to evaluate treatment related risk factors (S3 Table). This table displays the outcomes of the risk factor analyses for four of the types of hospitalization related health conditions with the highest relative hospitalization rates in CCS as compared to the reference population. Risk factor analyses were conducted among CCS in which treatment details were known (n = 5,607). Abbreviations: 95% CI: 95% confidence interval, CCS: Childhood Cancer Survivors, RHR: relative Hospitalization Ratio. ^1^ Groups are mutually exclusive.
(DOCX)

S8 Table Multivariable risk factor analyses for the effect of primary cancer type on the number of hospitalizations among childhood cancer survivors. * Other tumors comprise (frequency tables are displayed in S1 Table):
Germ cell tumors, trophoblastic tumors, and neoplasms of gonads (Gonadal carcinomas, Malignant gonadal germ cell tumors, Malignant extracranial and extragonadal germ cell tumors, Intracranial and intraspinal germ cell tumors, Other and unspecified malignant gonadal tumors)Other malignant epithelial neoplasms and malignant melanomas (Other and unspecified carcinomas, Skin carcinomas, Malignant melanomas, Nasopharyngeal carcinomas, Thyroid carcinomas, Adrenocortical carcinomas)Langerhans cell histiocytosisHepatic tumors (Hepatic carcinomas, Hepatoblastoma)RetinoblastomaOther and unspecified malignant neoplasms
(DOCX)

S9 Table Clinical characteristics in childhood cancer survivors by attained age groups. (DOCX)

S1 Checklist The RECORD statement—Checklist of items, extended from the STROBE statement, that should be reported in observational studies using routinely collected health data. (DOCX)

## Abbreviations

AER: absolute excess rate
CBS: Statistics Netherlands
CCS: childhood cancer survivors
CI: confidence interval
Dutch LATER: Dutch Childhood Oncology Group—Long term Effects after Childhood Cancer
GBA: Municipal Personal Records Database
GEE: Generalized Estimated Equations
ICD-9: International Classification of Diseases version 9
ICD-10: International Classification of Diseases version 10
IQR: interquartile range
LBZ: Dutch Hospital Discharge Register
POP: reference population
PY: person years
RHR: relative hospitalization rate
RIN: record identification number
RT: radiotherapy
