# Supplementary figures and images for: A detailed insight in the high risks of hospitalizations in long-term childhood cancer survivors—A Dutch LATER linkage study
Source: PLoS One. 2020 May 19;15(5):e0232708. doi: 10.1371/journal.pone.0232708 (PMC7236987; doi:10.1371/journal.pone.0232708)

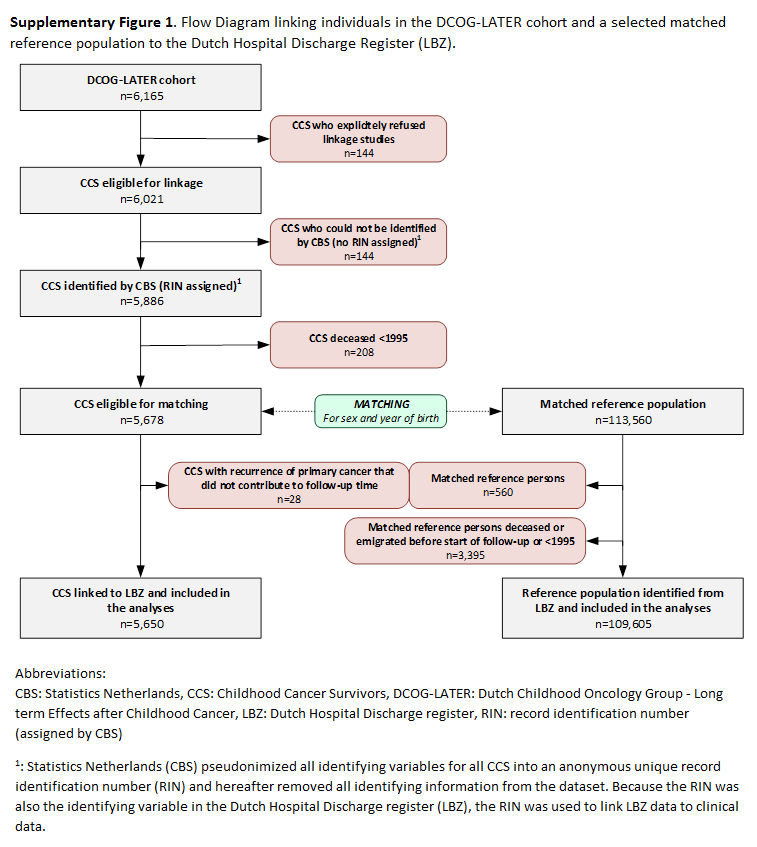

Supplement: S1 Fig — Abbreviations: CBS: Statistics Netherlands, CCS: Childhood Cancer Survivors, Dutch LATER: Dutch Childhood Oncology Group—Long term Effects after Childhood Cancer, LBZ: Dutch Hospital Discharge register, RIN: record identification number (assigned by CBS). 1: Statistics Netherlands (CBS) pseudonimized all identifying variables for all CCS into an anonymous unique record identification number (RIN) and hereafter removed all identifying information from the dataset. Because the RIN was also the identifying variable in the Dutch Hospital Discharge register (LBZ), the RIN was used to link LBZ data to clinical data. (TIF) [file pone.0232708.s001.tif]
