# Supplementary material for: A detailed insight in the high risks of hospitalizations in long-term childhood cancer survivors—A Dutch LATER linkage study
Source: PLoS One. 2020 May 19;15(5):e0232708. doi: 10.1371/journal.pone.0232708 (PMC7236987; doi:10.1371/journal.pone.0232708)
Supplement: S1 Table — (DOCX) [file pone.0232708.s002.docx]

**Supplementary Table S1.** Definition of variables

|  | **CCS study population (n=5,650)** | |
| --- | --- | --- |
| **Tumors** |  |  |
| *Leukemia* | 1,900 | 33.6% |
| *Hodgkin lymphoma* | 383 | 6.8% |
| *Non-Hodgkin lymphoma* | 543 | 9.6% |
| Mature B cell lymphoma (except Burkitt lymphoma) | 45 | 8.3% |
| Mature T cell and NK cell lymphomas | 64 | 11.8% |
| Non Hodgkin lymphomas NOS | 164 | 30.2% |
| Precursor cell lymphomas | 70 | 12.9% |
| Burkitt Lymphoma | 151 | 27.8% |
| Miscellaneous symphoreticular neoplasms and Unspecified lymphomas | 49 | 9.0% |
| *CNS tumor* | 744 | 13.2% |
| Ependymomas and choroid plexus tumor | 73 | 9.8% |
| Astrocytomas | 421 | 56.6% |
| Intracranial and intraspinal embryonal tumors | 177 | 23.8% |
| Other gliomas | 54 | 7.3% |
| Other specified and unspecified intracranial and intraspinal neoplasms | 19 | 2.6% |
| *Bone tumor* | 332 | 5.9% |
| Osteosarcomas | 181 | 54.5% |
| Ewing tumor and related sarcomas of bone | 129 | 38.9% |
| Chondrosarcomas, Other specified malignant bone tumors, Unspecified bone tumors | 22 | 6.6% |
| *Soft tissue sarcoma* | 406 | 7.2% |
| Rhabdomyosarcomas | 278 | 68.5% |
| Fibrosarcomas, peripheral nerve sheath tumors, and other fibromatous neoplasms | 31 | 7.6% |
| Other specified soft tissue sarcomas | 86 | 21.2% |
| Unspecified soft tissue sarcomas | 11 | 2.7% |
| *Renal tumor* | 567 | 10.0% |
| *Neuroblastoma* | 303 | 5.4% |
| *Other tumors* | 472 | 8.4% |
| Retinoblastoma | 30 | 6.4% |
| Hepatic tumors: hepatoblastoma/hepatic carcinoma | 49 | 10.4% |
| Germ cell tumors, trophoblastic tumors, and neoplasms of gonads | 217 | 46.0% |
| *Intracranial and intraspinal germ cell tumors* | *48* | *22.1%* |
| *Malignant extra cranial and extra gonadal germ cell tumors* | *51* | *23.5%* |
| *Malignant gonadal germ cell tumors* | *106* | *48.8%* |
| *Gonadal carcinomas and Other and unspecified malignant gonadal tumors* | *12* | *5.5%* |
| Other malignant epithelial neoplasms and malignant melanomas | 90 | 19.1% |
| *Thyroid carcinomas* | *24* | *26.7%* |
| *Nasopharyngeal carcinomas* | *17* | *18.9%* |
| *Malignant melanomas* | *21* | *23.3%* |
| *Adrenocortical carcinomas, Skin carcinomas and Other and unspecified carcinomas* | *28* | *31.1%* |
| Langerhans cell histiocytosis | 80 | 16.9% |
| Other and unspecified malignant neoplasms | <10 |  |
| **Chemotherapy** | 4,604 | 81.5% |
| *Alkylating agents* | 2,878 | 62.5% |
| Chlormethine/Mechlorethamine | 289 | 10.0% |
| Carmustine/BCNU | 18 | 0.6% |
| Nimustine/ACNU | <10 |  |
| Busulfan | 69 | 2.4% |
| *Busulfan intravenous administration* | *35* |  |
| *Busulfan oral administration* | *<10* |  |
| *Busulfan unknown route of administration* | *31* |  |
| Chlorambucil | <10 |  |
| Cyclophosphamide | 2,129 | 74.0% |
| *Cyclophosphamide intravenous administration* | *1,990* |  |
| *Cyclophosphamide oral administration* | *27* |  |
| *Cyclophosphamide unknown route of administration* | *142* |  |
| Dacarbazine | 161 | 5.6% |
| Ifosfamide | 661 | 23.0% |
| Lomustine | 75 | 2.6% |
| *Lomustine intravenous administration* | *31* |  |
| *Lomustine unknown route of administration* | *<10* |  |
| *Lomustine oral administration* | *42* |  |
| Melphalan | 82 | 2.8% |
| Procarbazine | 389 | 13.5% |
| *Procarbazine intravenous administration* | *84* |  |
| *Procarbazine unknown route of administration* | *<10* |  |
| *Procarbazine oral administration* | *302* |  |
| *Anthracyclines* | 2,605 | 56.6% |
| Daunorubicin | 1,045 | 40.1% |
| Doxorubicin | 1,821 | 69.9% |
| Epirubicin | 333 | 12.8% |
| Idarubicin | 66 | 2.5% |
| Mitoxantrone | 141 | 5.4% |
| *Platinum compounds* | 736 | 16.0% |
| Carboplatin | 389 | 52.9% |
| Cisplatin | 415 | 56.4% |
| Oxaliplatin | <10 |  |
| *Vinca alkaloids* | 4,074 | 88.5% |
| Vinblastine | 285 | 7.0% |
| Vincristine | 3,951 | 97.0% |
| Vinorelbine | <10 |  |
| Vindesine | 111 | 2.7% |
| *Antimetabolites* | 2,681 | 58.2% |
| Hydroxycarbamide | 25 | 0.9% |
| Methotrexate | 2,396 | 89.4% |
| *Methotrexate intravenous administration* | *1,979* |  |
| *Methotrexate intrathecal administration* | *2,093* |  |
| *Methotrexate unknown route of administration* | *61* |  |
| *Methotrexate oral administration* | *1,433* |  |
| Cladribine | <10 |  |
| Fludarabine | 15 | 0.6% |
| Mercaptopurine | 1,819 | 67.8% |
| *Mercaptopurine intravenous administration* | *68* |  |
| *Mercaptopurine unknown route of administration* | *26* |  |
| *Mercaptopurine oral administration* | *1,775* |  |
| Thioguanine | 710 | 26.5% |
| *Thioguanine intravenous administration* | *129* |  |
| *Thioguanine unknown route of administration* | *<10* |  |
| *Thioguanine oral administration* | *580* |  |
| Cytarabine | 2,043 | 76.2% |
| *Cytarabine intrathecal administration* | *1,639* |  |
| *Cytarabine intravenous administration* | *1,457* |  |
| *Cytarabine unknown route of administration* | *67* |  |
| Gemcitabine | <10 |  |
| *Epipodophyllotoxins* | 1,180 | 25.6% |
| Teniopside | 368 | 31.2% |
| Etoposide | 900 | 76.3% |
