# Supplementary material for: A detailed insight in the high risks of hospitalizations in long-term childhood cancer survivors—A Dutch LATER linkage study
Source: PLoS One. 2020 May 19;15(5):e0232708. doi: 10.1371/journal.pone.0232708 (PMC7236987; doi:10.1371/journal.pone.0232708)
Supplement: S2 Table — Abbreviations: 95% CI: 95% confidence interval, AER: Absolute Access Risk, CCS: Childhood Cancer Survivors, POP: reference population, PY: Person-Year, RHR: relative Hospitalization Ratio. Relative Hospitalization Ratios were adjusted for matched cases and controls, and for multiple hospitalizations. (DOCX) [file pone.0232708.s003.docx]

**Supplementary Table S2.** Hospitalizations in five year childhood cancer survivors and in the reference population, relative hospitalization risks and absolute access risks for overall hospitalizations and for hospitalization associated health condition type.

|  | **Number of hospitalizations in CCS (n)** | **Hospitalization rate in CCS (per 1,000 PY)** | **Number of hospitalizations in POP (n)** | **Hospitalization rate in POP (per 1,000 PY)** | **Adjusted RHR** | **(95% CI)** | **p-value** | **AER per 1,000 PY** |
| --- | --- | --- | --- | --- | --- | --- | --- | --- |
| **Overall hospitalization** | **16,141** | **177.86** | **122,490** | **77.68** | **2.01** | **(1.89-2.15)** | **<0.001** | **100.18** |
| IV - Endocrine, nutritional and metabolic diseases | 711 | 7.83 | 1,875 | 1.19 | **5.97** | **(4.61-7.73)** | **<0.001** | 6.65 |
| II - Neoplasms | 1,905 | 20.99 | 5,632 | 3.57 | **5.59** | **(4.64-6.73)** | **<0.001** | 17.42 |
| XVII - Symptoms, signs and abnormal clinical and laboratory findings, not elsewhere classified | 2,722 | 29.99 | 8,741 | 5.54 | **5.15** | **(4.57-5.82)** | **<0.001** | 24.45 |
| XII - Diseases of the skin and subcutaneous tissue | 446 | 4.91 | 2,732 | 1.73 | **2.90** | **(2.01-4.18)** | **<0.001** | 3.18 |
| IX - Diseases of the circulatory system | 614 | 6.77 | 3,747 | 2.38 | **2.87** | **(2.41-3.41)** | **<0.001** | 4.39 |
| XIX - Injury, poisoning and certain other consequences of external causes | 2,703 | 29.78 | 17,396 | 11.03 | **2.83** | **(2.42-3.30)** | **<0.001** | 18.75 |
| VII - Diseases of the eye and adnexa | 210 | 2.31 | 1,217 | 0.77 | **2.75** | **(1.99-3.79)** | **<0.001** | 1.54 |
| III - Diseases of the blood and blood-forming organs and certain disorder involving the immune mechanism | 196 | 2.16 | 1,340 | 0.85 | **2.70** | **(1.39-5.26)** | **0.003** | 1.31 |
| VI - Diseases of the nervous system | 559 | 6.16 | 3,724 | 2.36 | **2.68** | **(1.85-3.89)** | **<0.001** | 3.80 |
| VXII - Congenital malformations, deformations and chromosomal abnormalities | 185 | 2.04 | 1,321 | 0.84 | **2.31** | **(1.81-2.96)** | **<0.001** | 1.20 |
| I - Certain infectious and parasitic disorders | 118 | 1.30 | 1,015 | 0.64 | **2.20** | **(1.61-3.01)** | **<0.001** | 0.66 |
| VIII - Diseases of the ear and mastoid process | 258 | 2.84 | 2,346 | 1.49 | **2.04** | **(1.63-2.56)** | **<0.001** | 1.36 |
| XXI - Factors influencing health status and contact with health services | 299 | 3.29 | 2,639 | 1.67 | **1.95** | **(1.05-3.65)** | **0.034** | 1.62 |
| XIV - Diseases of the genitourinary system | 781 | 8.61 | 7,378 | 4.68 | **1.84** | **(1.55-2.17)** | **<0.001** | 3.93 |
| X - Diseases of the respiratory system | 667 | 7.35 | 7,554 | 4.79 | **1.59** | **(1.33-1.90)** | **<0.001** | 2.56 |
| XI - Diseases of the digestive system | 1,121 | 12.35 | 12,225 | 7.75 | **1.57** | **(1.29-1.92)** | **<0.001** | 4.60 |
| V - Mental and behavioral disorders | 100 | 1.10 | 1,235 | 0.78 | **1.51** | **(1.03-2.21)** | **<0.001** | 0.32 |
| XIII - Diseases of the musculoskeletal system and connective tissue | 807 | 8.89 | 12,879 | 8.17 | 1.08 | (0.95-1.23) | 0.225 | 0.73 |
| XVI - Certain conditions originating in the perinatal period | <10 | NA | NA | NA | NA | NA | NA | NA |

Abbreviations: 95% CI: 95% confidence interval, AER: Absolute Access Risk, CCS: Childhood Cancer Survivors, POP: reference population, PY: Person-Year, RHR: relative Hospitalization Ratio.
Relative Hospitalization Ratios were adjusted for matched cases and controls, and for multiple hospitalizations.
