# Supplementary material for: A detailed insight in the high risks of hospitalizations in long-term childhood cancer survivors—A Dutch LATER linkage study
Source: PLoS One. 2020 May 19;15(5):e0232708. doi: 10.1371/journal.pone.0232708 (PMC7236987; doi:10.1371/journal.pone.0232708)
Supplement: S3 Table — (DOCX) [file pone.0232708.s004.docx]

**Supplementary Table S3.** Specification of underlying types of health conditions

|  | **CCS study population (n=5,650)** | |  | **Reference population (n=109,605)** | |
| --- | --- | --- | --- | --- | --- |
| **IV - Endocrine, nutritional and metabolic diseases** | 364 | 6.4% |  | 1002 | 0.9% |
| Nutritional & metabolic disorders | 89 | 24.5% |  | 513 | 51.2% |
| *Metabolic disorders* | 87 | 97.8% |  | 504 | 98.2% |
| Disorders of adrenal gland | 68 | 18.7% |  | 22 | 2.2% |
| Disorders of thyroid gland | 51 | 14.0% |  | 117 | 11.7% |
| *Goiter* | 25 | 49.0% |  | 41 | 35.0% |
| *Hypothyroidism* | 12 | 23.5% |  |  |  |
| *Other thyroid disorder (including thyrotoxicosis, thyroiditis, etc)* | 15 | 29.4% |  | 72 | 61.5% |
| Other endocrine conditions (including parathyroid disorders, thymus disorders, gonadal disorders, etc) | 30 | 8.2% |  | 32 | 3.2% |
| Disorders of pituitary gland and its hypothalamic control | 21 | 5.8% |  |  | 0.0% |
| Diabetes mellitus and other disorders of pancreatic secretion | 19 | 5.2% |  | 312 | 31.1% |
| **II - Neoplasms** | 637 | 11.3% |  | 2397 | 2.2% |
| Malignant neoplasms |  |  |  |  |  |
| *Malignant neoplasm of bone, connective tissue, skin and breast* | 139 | 21.8% |  | 297 | 12.4% |
| *Malignant neoplasm of skin* | 58 | 9.1% |  | 90 | 3.8% |
| *Malignant neoplasm of breast* | 43 | 6.8% |  | 159 | 6.6% |
| *Malignant neoplasm of bone, connective tissue and other soft tissue* | 52 | 8.2% |  | 115 | 4.8% |
| *Other malignant neoplasm (including malignant neoplasm of brain, other parts of nervous system, eye, etc)* | 127 | 19.9% |  | 219 | 9.1% |
| *Malignant neoplasm of lymphatic and hematopoietic tissue* | 44 | 6.9% |  | 130 | 5.4% |
| *Malignant neoplasm of digestive organs and peritoneum* | 23 | 3.6% |  | 92 | 3.8% |
| *Malignant neoplasm of genitourinary organs* | 22 | 3.5% |  | 161 | 6.7% |
| *Malignant neoplasm of throat and respiratory and intrathoracic organs* | 15 | 2.4% |  | 49 | 2.0% |
| Benign neoplasms | 299 | 46.9% |  | 1372 | 57.2% |
| Neoplasms of uncertain behavior or unspecified nature | 99 | 15.5% |  | 158 | 6.6% |
| Carcinoma in situ | 13 | 2.0% |  | 132 | 5.5% |
| **XII - Diseases of the skin and subcutaneous tissue** | 221 | 3.9% |  | 1725 | 1.6% |
| Other diseases of skin and subcutaneous tissue | 136 | 61.5% |  | 748 | 43.4% |
| Infections of skin and subcutaneous tissue | 69 | 31.2% |  | 865 | 50.1% |
| Other inflammatory conditions of skin and subcutaneous tissue | 21 | 9.5% |  | 157 | 9.1% |
| **IX - Diseases of the circulatory system** | 296 | 5.2% |  | 2454 | 2.2% |
| Other forms of heart disease (including acute rheumatic fever, chronic rheumatic heart disease, etc.) | 110 | 37.2% |  | 543 | 22.1% |
| Other diseases of veins and lymphatics, and other diseases of circulatory system | 79 | 26.7% |  | 1060 | 43.2% |
| Cerebrovascular disease | 67 | 22.6% |  | 200 | 8.1% |
| Ischemic heart disease | 32 | 10.8% |  | 344 | 14.0% |
| Diseases of pulmonary circulation | 16 | 5.4% |  | 142 | 5.8% |
| Diseases of arteries, arterioles and capillaries | 16 | 5.4% |  | 129 | 5.3% |
| Hypertensive disease | 12 | 4.1% |  | 90 | 3.7% |
| **XIX - Injury, poisoning and certain other consequences of external causes** | 1226 | 21.7% |  | 10987 | 10.0% |
| Including fractures, dislocation, sprains and spraints of joints, intracranial injury, internal injury of thorax/abdomen/pelvis, open wounds, etc.) | |  |  |  |  |
| **VII - Diseases of the eye and adnexa** | 132 | 2.3% |  | 875 | 0.8% |
| Other disorders of eye and adnexa | 84 | 63.6% |  | 542 | 61.9% |
| Cataract and glaucoma | 26 | 19.7% |  | 139 | 15.9% |
| *Cataract* | 23 | 17.4% |  | 118 | 13.5% |
| **III - Diseases of the blood and blood-forming organs and certain disorder involving the immune mechanism** | 71 | 1.3% |  | 378 | 0.3% |
| Anemias | 41 | 31.1% |  | 220 | 25.1% |
| Other diseases of blood and blood-forming organs | 36 | 27.3% |  | 182 | 20.8% |
| Coagulation defects, purpura and other hemorrhagic conditions | 12 | 9.1% |  | 143 | 16.3% |
| **VI - Diseases of the nervous system** | 265 | 4.7% |  | 1768 | 1.6% |
| Including diseases of the central nervous system, disorders of the peripheral nervous system, spinal disorders, etc.) |  |  |  |  |  |
| **I - Certain infectious and parasitic disorders** | 91 | 1.6% |  | 796 | 0.7% |
| Including specific infectious and parasitic diseases |  |  |  |  |  |
| **VIII - Diseases of the ear and mastoid process** | 160 | 2.8% |  | 1568 | 1.4% |
| Diseases of middle ear and mastoid | 116 | 72.5% |  | 1177 | 75.1% |
| Other disorders of ear | 17 | 10.6% |  | 148 | 9.4% |
| Diseases of external ear | 13 | 8.1% |  | 52 | 3.3% |
| Diseases of inner ear | 10 | 6.3% |  | 77 | 4.9% |
| **XXI - Factors influencing health status and contact with health services** | 205 | 3.6% |  | 1684 | 1.5% |
| Including: persons encountering health services for examination and investigation, persons with potential health hazards related to communicable diseases, | | | |  |  |
| persons encountering health services in circumstances related to reproduction, persons encountering health services for specific procedures and health care, | | | |  |  |
| persons with potential health hazards related to socioeconomic and psychosocial circumstances, persons encountering health services in other circumstances, | | | | |  |
| persons with potential health hazards related to family and personal history and certain conditions influencing health status |  |  |  |  |  |
| **XIV - Diseases of the genitourinary system** | 473 | 8.4% |  | 5167 | 4.7% |
| Diseases of the male and female genital tract | 261 | 55.2% |  | 3412 | 66.0% |
| Renal diseases | 140 | 29.6% |  | 1076 | 20.8% |
| Disorders of breast | 99 | 20.9% |  | 821 | 15.9% |
| **X - Diseases of the respiratory system** | 449 | 7.9% |  | 6220 | 5.7% |
| Other diseases of the upper respiratory tract | 283 | 63.0% |  | 5186 | 83.4% |
| *Diseases of nose or pharynx* | 67 | 23.7% |  | 1257 | 24.2% |
| *Chronic sinusitis* | 49 | 17.3% |  | 585 | 11.3% |
| *Diseases of tonsils (including tonsillitis, peritonsillair abscess, etc.)* | 135 | 47.7% |  | 3092 | 59.6% |
| *Other upper respiratory (including conditions of larynx, etc.)* | 49 | 17.3% |  | 539 | 10.4% |
| Pneumonia and influenza | 80 | 17.8% |  | 412 | 6.6% |
| Other diseases of the respiratory system (including lung diseases due to external agents) | 67 | 14.9% |  | 339 | 5.5% |
| *Other diseases of the respiratory system* | 58 | 86.6% |  | 331 | 97.6% |
| Chronic obstructive pulmonary disease and allied conditions | 25 | 5.6% |  | 251 | 4.0% |
| Acute respiratory infections | 24 | 5.3% |  | 172 | 2.8% |
| **XI - Diseases of the digestive system** | 635 | 11.2% |  | 7729 | 7.1% |
| Diseases of intestines and peritoneum | 190 | 29.9% |  | 2059 | 26.6% |
| Diseases of oral cavity, salivary glands and jaws | 164 | 25.8% |  | 1381 | 17.9% |
| Appendicitis | 105 | 16.5% |  | 1803 | 23.3% |
| Diseases of liver and biliary tract | 76 | 12.0% |  | 776 | 10.0% |
| Other diseases of digestive system | 72 | 11.3% |  | 1439 | 18.6% |
| Diseases of esophagus, stomach and duodenum | 57 | 9.0% |  | 418 | 5.4% |
| **V - Mental and behavioral disorders** | 73 | 1.3% |  | 883 | 0.8% |
| Including psychoses, neurotic disorders, personality disorders, other nonpsychotic mental disorders, mental retardation, etc. |  |  |  |  |  |
| **XIII - Diseases of the musculoskeletal system and connective tissue** | 507 | 9.0% |  | 7897 | 7.2% |
| Including arthropathies and related disorders, dorsopathies, rheumatism, osteopathies, chondropathies, acquired musculoskeletal deformities | |  |  |  |  |
| **XV - Pregnancy** | 567 | 10.0% |  | 10916 | 10.0% |
| Including ectopic and molar pregnancy, other pregnancy with abortive outcome, complications related to pregnancy, complications of labor/puerperium, etc. | | | |  |  |
