# Supplementary material for: A detailed insight in the high risks of hospitalizations in long-term childhood cancer survivors—A Dutch LATER linkage study
Source: PLoS One. 2020 May 19;15(5):e0232708. doi: 10.1371/journal.pone.0232708 (PMC7236987; doi:10.1371/journal.pone.0232708)
Supplement: S4 Table — 1: Hospitalization rate in reference population: 77.68/1,000 PY Abbreviations: 95% CI: 95% confidence interval, AER: Absolute Access Risk, CCS: Childhood Cancer Survivors, POP: reference population, PY: Person-Year, RHR: relative Hospitalization Ratio. Relative Hospitalization Ratios were adjusted for matched cases and controls, and for multiple hospitalizations. *Other tumors comprise (for frequency table, see S1 Table): Germ cell tumors, trophoblastic tumors, and neoplasms of gonads (Gonadal carcinomas, Malignant gonadal germ cell tumors, Malignant extra cranial and extra gonadal germ cell tumors, Intracranial and intraspinal germ cell tumors, Other and unspecified malignant gonadal tumors)Other malignant epithelial neoplasms and malignant melanomas (Other and unspecified carcinomas, Skin carcinomas, Malignant melanomas, Nasopharyngeal carcinomas, Thyroid carcinomas, Adrenocortical carcinomas)Langerhans cell histiocytosisHepatic tumors (Hepatic carcinomas, Hepatoblastoma)RetinoblastomaOther and unspecified malignant neoplasms (DOCX) [file pone.0232708.s005.docx]

**Supplementary Table S4.** Hospitalizations in five-year childhood cancer survivors and in the reference population, relative hospitalization risks and absolute access risks, per childhood cancer diagnosis

|  | **Number of hospitalizations in CCS (n)** | **Total years of follow-up in CCS** | **Hospitalization rate in CCS (per 1000 PY)^1^** | **Adjusted RHR** | **(95% CI)** | **p-value** | **AER per 1000 PY** |
| --- | --- | --- | --- | --- | --- | --- | --- |
| Other tumors* | 1966 | 7752.15 | 253.61 | **2.69** | **2.07-3.49** | **<0.001** | 175.93 |
| Bone tumors | 1283 | 5322.28 | 241.06 | **2.29** | **1.89-2.76** | **<0.001** | 163.38 |
| Central nervous system tumors | 2405 | 10676.92 | 225.25 | **2.19** | **1.96-2.44** | **<0.001** | 147.57 |
| Soft tissue sarcomas | 1373 | 6515.79 | 210.72 | **2.14** | **1.77-2.57** | **<0.001** | 133.04 |
| Leukemia | 4632 | 30197.82 | 153.39 | **1.87** | **1.65-2.12** | **<0.001** | 75.71 |
| *ALL* | 3861 | 26287.48 | 146.88 | 1.80 | **1.56-2.07** | **<0.001** | 69.20 |
| *AML* | 573 | 2888.49 | 198.37 | 2.24 | **1.67-3.11** | **<0.001** | 120.70 |
| *Other leukemia* | 198 | 1021.85 | 193.77 | 2.53 | **1.61-3.98** | **<0.001** | 116.09 |
| Hodgkin lymphomas | 908 | 6037.68 | 150.39 | **1.84** | **1.47-2.31** | **<0.001** | 72.71 |
| Renal tumors | 1627 | 10183.69 | 159.77 | **1.84** | **1.47-2.29** | **<0.001** | 82.09 |
| Neuroblastomas | 825 | 5057.47 | 163.13 | **1.74** | **1.45-2.09** | **<0.001** | 85.45 |
| Non-Hodgkin lymphomas | 1122 | 9008.34 | 124.55 | **1.68** | **1.28-2.21** | **<0.001** | 46.87 |

^1^: Hospitalization rate in reference population: 77.68/1,000 PY
Abbreviations: 95% CI: 95% confidence interval, AER: Absolute Access Risk, CCS: Childhood Cancer Survivors, POP: reference population, PY: Person-Year, RHR: relative Hospitalization Ratio.
Relative Hospitalization Ratios were adjusted for matched cases and controls, and for multiple hospitalizations.

*Other tumors comprise (for frequency table, see Supplementary Table S1):

- Germ cell tumors, trophoblastic tumors, and neoplasms of gonads (*Gonadal carcinomas, Malignant gonadal germ cell tumors, Malignant extra cranial and extra gonadal germ cell tumors, Intracranial and intraspinal germ cell tumors, Other and unspecified malignant gonadal tumors*)

- Other malignant epithelial neoplasms and malignant melanomas (*Other and unspecified carcinomas, Skin carcinomas, Malignant melanomas, Nasopharyngeal carcinomas, Thyroid carcinomas, Adrenocortical carcinomas*)

- Langerhans cell histiocytosis

- Hepatic tumors (*Hepatic carcinomas, Hepatoblastoma*)

- Retinoblastoma

- Other and unspecified malignant neoplasms
