# Supplementary material for: A detailed insight in the high risks of hospitalizations in long-term childhood cancer survivors—A Dutch LATER linkage study
Source: PLoS One. 2020 May 19;15(5):e0232708. doi: 10.1371/journal.pone.0232708 (PMC7236987; doi:10.1371/journal.pone.0232708)
Supplement: S5 Table — Chi square: p<0.001 Abbreviations: CCS: childhood cancer survivors, POP: reference population. (DOCX) [file pone.0232708.s006.docx]

**Supplementary Table S6.** Frequency table of the total number of hospitalizations per person for hospitalizations because of symptoms without an underlying diagnosis among childhood cancer survivors and among the reference population

| **No. hospitalizations because of symptoms without an underlying diagnosis** | **CCS (n=5,650)** | | **Reference population (n=109,605)** | |
| --- | --- | --- | --- | --- |
| **0** | 4,462 | (78.97%) | 103,710 | (94.62%) |
| **1** | 730 | (12.92%) | 4554 | (4.15%) |
| **2** | 223 | (3.95%) | 846 | (0.77%) |
| **3** | 87 | (1.54%) | 267 | (0.24%) |
| **4** | 41 | (0.73%) | 95 | (0.09%) |
| **5** | 31 | (0.55%) | 47 | (0.04%) |
| **6-10** | 44 | (0.78%) | 64 | (0.06%) |
| **>10** | 30 | (0.53%) | 22 | (0.02%) |

Chi square: p<0.001
Abbreviations: CCS: childhood cancer survivors, POP: reference population.
