# Supplementary material for: A detailed insight in the high risks of hospitalizations in long-term childhood cancer survivors—A Dutch LATER linkage study
Source: PLoS One. 2020 May 19;15(5):e0232708. doi: 10.1371/journal.pone.0232708 (PMC7236987; doi:10.1371/journal.pone.0232708)
Supplement: S6 Table — Chi square: p<0.001 Abbreviations: CCS: childhood cancer survivors. Frequencies of all hospitalizations for each specific ICD-10 code were listed, specific ICD-10 codes were grouped into categories of health conditions and presented in this table. This table sums the total number of hospitalizations and not the number of individual; one individual can contribute multiple hospitalizations. (DOCX) [file pone.0232708.s007.docx]

**Supplementary Table S5.** Summary of types of discharge diagnosis for all hospital admissions because of symptoms, signs and abnormal clinical findings among childhood cancer survivors and among the reference population

|  | **CCS**  (n=2,722 hospitalizations because of symptoms without an underlying diagnosis) | | **Reference population**  (n=8,471 hospitalizations because of symptoms without an underlying diagnosis) | |
| --- | --- | --- | --- | --- |
| Symptoms of the circulatory system | 26 | (0.96%) | 193 | (2.28%) |
| Symptoms of the digestive system | 236 | (8.67%) | 2,168 | (25.59%) |
| General symptoms | 319 | (11.72%) | 1,423 | (16.80%) |
| Symptoms of head and neck | 58 | (2.13%) | 354 | (4.18%) |
| Symptoms of the nervous and musculoskeletal system | 13 | (0.48%) | 61 | (0.72%) |
| Symptoms of the respiratory system | 147 | (5.40%) | 1,782 | (21.04%) |
| Symptoms of the skin | 14 | (0.51%) | 82 | (0.97%) |
| Abnormal test results | 25 | (0.92%) | 144 | (1.70%) |
| Symptoms of the urinary tract | 21 | (0.77%) | 179 | (2.11%) |
| Other symptoms and symptoms unknown | 1,863 | (68.44%) | 2,058 | (24.29%) |

Chi square: p<0.001
Abbreviations: CCS: childhood cancer survivors.

Frequencies of all hospitalizations for each specific ICD-10 code were listed, specific ICD-10 codes were grouped into categories of health conditions and presented in this table. This table sums the total number of hospitalizations and not the number of individual; one individual can contribute multiple hospitalizations.
