# Supplementary material for: A detailed insight in the high risks of hospitalizations in long-term childhood cancer survivors—A Dutch LATER linkage study
Source: PLoS One. 2020 May 19;15(5):e0232708. doi: 10.1371/journal.pone.0232708 (PMC7236987; doi:10.1371/journal.pone.0232708)
Supplement: S7 Table — For each category of hospitalization related health conditions, a separate Poisson regression model was performed to evaluate treatment related risk factors (S3 Table). This table displays the outcomes of the risk factor analyses for four of the types of hospitalization related health conditions with the highest relative hospitalization rates in CCS as compared to the reference population. Risk factor analyses were conducted among CCS in which treatment details were known (n = 5,607). Abbreviations: 95% CI: 95% confidence interval, CCS: Childhood Cancer Survivors, RHR: relative Hospitalization Ratio. 1 Groups are mutually exclusive. (DOCX) [file pone.0232708.s008.docx]

**Supplementary Table S7**. Multivariable risk factor analyses for the effect of treatment related risk factors on the number of hospitalizations among childhood cancer survivors.

For each category of hospitalization related health conditions, a separate Poisson regression model was performed to evaluate treatment related risk factors (Supplementary Table 3). This table displays the outcomes of the risk factor analyses for four of the types of hospitalization related health conditions with the highest relative hospitalization rates in CCS as compared to the reference population. Risk factor analyses were conducted among CCS in which treatment details were known (n=5,607)

|  | **XIX - Injury, poisoning and certain other consequences of external causes** | | | | **VII - Diseases of the eye and adnexa** | | | | **III - Diseases of the blood and blood-forming organs and certain disorder involving the immune mechanism** | | | | **VI - Diseases of the nervous system** | | | | **I - Certain infectious and parasitic disorders** | | | |
| --- | --- | --- | --- | --- | --- | --- | --- | --- | --- | --- | --- | --- | --- | --- | --- | --- | --- | --- | --- | --- |
|  | **n/n with event** | RHR | 95%CI | p-value | **n/n with event** | RHR | 95%CI | p-value | **n/n with event** | RHR | 95%CI | p-value | **n/n with event** | RHR | 95%CI | p-value | **n/n with event** | RHR | 95%CI | p-value |
| **Sex^1^** |  |  |  |  |  |  |  |  |  |  |  |  |  |  |  |  |  |  |  |  |
| Male | 3125/618 | Ref |  |  | 3125/70 | Ref |  |  | 3125/33 | Ref |  |  | 3125/142 | Ref |  |  | 3125/45 | Ref |  |  |
| Female | 2482/600 | 1.078 | 0.839-1.387 | 0.557 | 2482/61 | 1.228 | 0.737-2.047 | 0.431 | 2482/38 | 0.965 | 0.358-2.599 | 0.944 | 2482/122 | 1.834 | 0.896-3.754 | 0.097 | 2482/45 | 1.287 | 0.805-2.057 | 0.292 |
| **Age at diagnosis,years^1^** |  |  |  |  |  |  |  |  |  |  |  |  |  |  |  |  |  |  |  |  |
| 0-4 | 2543/539 | Ref |  |  | 2543/57 | Ref |  |  | 2543/30 | Ref |  |  | 2543/100 | Ref |  |  | 2543/38 | Ref |  |  |
| 5-9 | 1519/338 | 0.766 | 0.535-1.097 | 0.146 | 1519/43 | 1.349 | 0.784-2.320 | 0.279 | 1519/15 | 0.437 | 0.141-1.354 | 0.151 | 1519/85 | 2.076 | 0.934-4.612 | 0.073 | 1519/28 | 1.294 | 0.747-2.242 | 0.358 |
| 10-14 | 1193/267 | 0.769 | 0.555-1.066 | 0.115 | 1193/23 | 0.661 | 0.364-1.199 | 0.173 | 1193/21 | 1.820 | 0.484-6.849 | 0.376 | 1193/63 | 1.099 | 0.710-1.702 | 0.672 | 1193/16 | 0.690 | 0.349-1.363 | 0.285 |
| 15-17 | 352/74 | 0.756 | 0.528-1.083 | 0.128 | 352/<10 | 0.714 | 0.323-1.578 | 0.406 | 352/<10 | 0.783 | 0.205-3.000 | 0.722 | 352/16 | 1.108 | 0.569-2.156 | 0.763 | 352/<10 | 1.356 | 0.568-3.238 | 0.493 |
| **Follow-up time** |  | 1.018 | 0.991-1.046 | 0.186 |  | **1.079** | **1.039-1.120** | **<0.001** |  | 1.027 | 0.951-1.109 | 0.492 |  | 1.043 | 1.000-1.089 | 0.052 |  | **1.070** | **1.022-1.119** | **0.003** |
| **Surgery** | 3797/845 | 1.501 | 0.836-2.696 | 0.174 | 3797/93 | 1.585 | 0.555-4.526 | 0.389 | 3797/56 | **5.355** | **1.766-16.236** | **0.003** | 3797/196 | 2.209 | 0.706-6.907 | 0.173 | 3797/68 | 1.761 | 0.856-3.625 | 0.124 |
| **Radiotherapy (RT)** |  |  |  |  |  |  |  |  |  |  |  |  |  |  |  |  |  |  |  |  |
| Cranial RT | 1193/336 | 1.501 | 1.000-2.255 | 0.050 | 1193/50 | **2.505** | **1.483-4.233** | **0.001** | 1193/19 | 0.433 | 0.159-1.184 | 0.103 | 1193/128 | **2.316** | **1.220-4.395** | **0.010** | 1193/22 | 1.437 | 0.755-2.736 | 0.269 |
| Spinal RT | 355/108 | 0.839 | 0.571-1.232 | 0.370 | 355/13 | 1.023 | 0.319-3.278 | 0.969 | 355/<10 | 1.622 | 0.616-4.274 | 0.328 | 355/32 | 0.609 | 0.366-1.015 | 0.057 | 355/<10 | 0.908 | 0.346-2.386 | 0.845 |
| Total body irradiat. | 200/66 | **3.336** | **1.527-7.289** | **0.003** | 200/<10 | 2.943 | 1.032-8.392 | 0.043 | 200/<10 | 3.327 | 0.513-21.581 | 0.208 | 200/<10 | 0.603 | 0.161-2.261 | 0.453 | 200/<10 | 2.007 | 0.727-5.541 | 0.179 |
| RT Thorax | 351/80 | 1.121 | 0.748-1.680 | 0.580 | 351/<10 | 0.833 | 0.417-1.665 | 0.606 | 351/<10 | 0.551 | 0.186-1.630 | 0.281 | 351/13 | 0.795 | 0.372-1.699 | 0.554 | 351/<10 | 0.659 | 0.262-1.660 | 0.376 |
| Abdominalpelvic RT | 420/92 | 0.876 | 0.659-1.163 | 0.359 | 420/<10 | 0.497 | 0.198-1.246 | 0.136 | 420/<10 | 0.940 | 0.228-3.879 | 0.932 | 420/<10 | **0.305** | **0.144-0.646** | **0.002** | 420/14 | **2.363** | **1.157-4.826** | **0.018** |
| Neck RT | 218/44 | 0.829 | 0.541-1.270 | 0.389 | 218/<10 | 1.337 | 0.589-3.038 | 0.488 | 218/<10 | 0.350 | 0.073-1.678 | 0.189 | 218/<10 | 0.558 | 0.219-1.420 | 0.221 | 218/<10 | 0.830 | 0.272-2.527 | 0.742 |
| RT Upper extremities | 41/<!0 | 1.915 | 0.880-4.165 | 0.101 | 41/0 | NA |  |  | 41/0 | NA |  |  | 41/<10 | 1.028 | 0.398-2.658 | 0.954 | 41/0 | NA |  |  |
| RT Lower extremities | 73/<10 | **2.493** | **1.331-4.670** | **0.004** | 73/<10 | 0.515 | 0.072-3.675 | 0.508 | 73/0 | NA |  |  | 73/<10 | 0.471 | 0.106-2.088 | 0.322 | 73/<10 | **5.597** | **2.010-15.583** | **0.001** |
| **Chemotherapy** |  |  |  |  |  |  |  |  |  |  |  |  |  |  |  |  |  |  |  |  |
| Anthracyclines | 2605/555 | 0.681 | 0.376-1.231 | 0.204 | 2605/45 | 0.818 | 0.497-1.346 | 0.430 | 2605/32 | 0.345 | 0.079-1.506 | 0.157 | 2605/64 | 0.588 | 0.287-1.202 | 0.145 | 2605/39 | 0.957 | 0.549-1.668 | 0.877 |
| Alkylating agents | 2878/641 | 1.357 | 0.836-2.202 | 0.217 | 2878/59 | 0.565 | 0.287-1.112 | 0.098 | 2878/42 | 1.970 | 0.490-7.930 | 0.340 | 2878/109 | 1.131 | 0.730-1.752 | 0.581 | 2878/49 | 1.261 | 0.721-2.205 | 0.417 |
| Platinum | 736/192 | 0.970 | 0.568-1.655 | 0.910 | 736/26 | 1.349 | 0.605-3.011 | 0.464 | 736/14 | 0.963 | 0.404-2.293 | 0.931 | 736/39 | 1.192 | 0.679-2.093 | 0.540 | 736/14 | 1.225 | 0.618-2.427 | 0.561 |
| Vinca alkaloids | 4074/839 | 0.895 | 0.664-1.206 | 0.466 | 4074/78 | 0.859 | 0.477-1.546 | 0.612 | 4074/42 | **0.426** | **0.203-0.893** | **0.024** | 4074/153 | 0.803 | 0.567-1.139 | 0.218 | 4074/62 | 0.950 | 0.524-1.723 | 0.867 |
| Antimetabolites | 2618/548 | 1.253 | 0.558-2.812 | 0.584 | 2618/52 | 1.012 | 0.379-2.704 | 0.981 | 2618/30 | 3.946 | 1.177-12.226 | 0.026 | 2618/96 | 2.031 | 0.783-5.271 | 0.145 | 2618/36 | 0.923 | 0.492-1.731 | 0.803 |
| Epipodophyllotoxins | 1180/270 | 1.190 | 0.730-1.939 | 0.485 | 1180/41 | **2.025** | **1.007-4.070** | **0.048** | 1180/18 | 0.958 | 0.447-2.051 | 0.911 | 1180/40 | 0.548 | .281-1.066 | 0.076 | 1180/22 | 1.222 | 0.677-2.206 | 0.506 |

Abbreviations: 95% CI: 95% confidence interval, CCS: Childhood Cancer Survivors, RHR: relative Hospitalization Ratio.
^1^ Groups are mutually exclusive

**Supplementary Table S6**. (continued)

|  | **VIII - Diseases of the ear and mastoid process** | | | | **XXI - Factors influencing health status and contact with health services** | | | | **XIV - Diseases of the genitourinary system** | | | | **X - Diseases of the respiratory system** | | | | **XI - Diseases of the digestive system** | | | |
| --- | --- | --- | --- | --- | --- | --- | --- | --- | --- | --- | --- | --- | --- | --- | --- | --- | --- | --- | --- | --- |
|  | **n/n with event** | RHR | 95%CI | p-value | **n/n with event** | RHR | 95%CI | p-value | **n/n with event** | RHR | 95%CI | p-value | **n/n with event** | RHR | 95%CI | p-value | **n/n with event** | RHR | 95%CI | p-value |
| **Sex^1^** |  |  |  |  |  |  |  |  |  |  |  |  |  |  |  |  |  |  |  |  |
| Male | 3125/86 | Ref |  |  | 3125/90 | Ref |  |  | 3125/173 | Ref |  |  | 3125/207 | Ref |  |  | 3125/299 | Ref |  |  |
| Female | 2482/72 | 0.960 | 0.645-1.429 | 0.841 | 2482/114 | **2.568** | **1.515-4.352** | **<0.001** | 2482/297 | **2.422** | **1.857-3.159** | **<0.001** | 2482/235 | 1.714 | 1.185-2.479 | **0.004** | 2482/224 | 1.328 | 0.947-1.863 | 0.100 |
| **Age at diagnosis,years^1^** |  |  |  |  |  |  |  |  |  |  |  |  |  |  |  |  |  |  |  |  |
| 0-4 | 2543/112 | Ref |  |  | 2543/90 | Ref |  |  | 2543/218 | Ref |  |  | 2543/207 | Ref |  |  | 2542/297 | Ref |  |  |
| 5-9 | 1519/26 | **0.309** | **0.181-0.528** | **<0.001** | 1519/46 | 0.861 | 0.577-1.284 | 0.462 | 1519/112 | 0.761 | 0.576-1.007 | 0.056 | 1519/113 | 1.192 | 0.733-1.938 | 0.480 | 1519/166 | 1.082 | 0.783-1.496 | 0.633 |
| 10-14 | 1193/13 | **0.287** | **0.126-0.652** | **0.003** | 1193/52 | 1.992 | 0.849-4.674 | 0.113 | 1193/110 | 1.320 | 0.932-1.870 | 0.117 | 1193/102 | 1.293 | 0.838-1.994 | 0.245 | 1193/133 | 1.471 | 0.872-2.479 | 0.148 |
| 15-17 | 352/<10 | 0.409 | 0.184-0.907 | 0.028 | 352/16 | 1.812 | 0.830-3.954 | 0.136 | 352/30 | 1.013 | 0.647-1.586 | 0.955 | 352/20 | 0.645 | 0.386-1.077 | 0.094 | 352/37 | 0.834 | 0.530-1.311 | 0.431 |
| **Follow-up time** |  | 1.038 | 1.02-1.076 | 0.037 |  | **1.155** | **1.086-1.228** | **<0.001** |  | **1.130** | **1.097-1.165** | **<0.001** |  | 1.041 | 1.08-1.075 | **0.015** |  | **1.051** | **1.018-1.085** | **0.002** |
| **Surgery** | 3797/113 | 1.382 | 0.790-2.415 | 0.256 | 3797/133 | 1.227 | 0.753-2.000 | 0.412 | 3797/344 | 0.998 | 0.715-1.392 | 0.989 | 3797/296 | 1.707 | 0.873-3.339 | 0.118 | 3797/436 | 0.703 | 0.261-1.897 | 0.486 |
| **Radiotherapy** |  |  |  |  |  |  |  |  |  |  |  |  |  |  |  |  |  |  |  |  |
| Cranial RT | 1193/39 | 1.003 | 0.592-1.699 | 0.991 | 1193/63 | 1.325 | 0.908-1.934 | 0.145 | 1193/116 | 1.038 | 0.767-1.405 | 0.809 | 1193/114 | 1.163 | 0.823-1.644 | 0.392 | 1193/161 | 1.012 | 0.680-1.504 | 0.954 |
| Spinal RT | 355/13 | 1.586 | 0.664-3.791 | 0.299 | 355/17 | 0.981 | 0.518-1.531 | 0.675 | 355/39 | 1.252 | 0.818-1.916 | 0.301 | 355/22 | **0.509** | **0.316-0.821** | **0.006** | 355/47 | 0.909 | 0.591-1.400 | 0.666 |
| Total body irradiat. | 200/<10 | 1.163 | 0.384-3.524 | 0.790 | 200/10 | 1.522 | 0.716-3.237 | 0.275 | 200/19 | 1.972 | 1.145-3.397 | 0.014 | 200/24 | **5.129** | **1.805-14.574** | **0.002** | 200/29 | 1.857 | 0.941-3.668 | 0.075 |
| RT Thorax | 351/<10 | 0.427 | 0.166-1.097 | 0.077 | 351/16 | 0.596 | 0.267-1.331 | 0.207 | 351/36 | 0.805 | 0.521-1.245 | 0.330 | 351/33 | 1.189 | 0.698-2.025 | 0.523 | 351/43 | 1.898 | 0.759-4.748 | 0.171 |
| Abdominalpelvic RT | 420/13 | 1.047 | 0.529-2.070 | 0.896 | 420/19 | 0.735 | 0.377-1.432 | 0.366 | 420/61 | **1.835** | **1.201-2.803** | **0.005** | 420/33 | 1.069 | 0.593-1.925 | 0.824 | 420/56 | 1.964 | 0.993-3.882 | 0.052 |
| Neck RT | 218/<10 | 4.167 | 1.425-12.190 | **0.009** | 218/10 | 0.878 | 0.363-2.126 | 0.773 | 218/14 | 0.585 | 0.305-1.123 | 0.107 | 218/23 | 1.411 | 0.821-2.426 | 0.213 | 218/26 | 0.492 | 0.154-1.579 | 0.234 |
| RT Upper extremities | 41/<10 | 0.931 | 0.144-6.025 | 0.940 | 41/<10 | 0.258 | 0.031-2.160 | 0.211 | 41/<10 | 0.533 | 0.135-2.022 | 0.347 | 41/<10 | 0.550 | 0.129-2.349 | 0.419 | 41/<10 | 0.403 | 0.144-1.125 | 0.083 |
| RT Lower extremities | 73/0 | NA |  |  | 73/<10 | 0.832 | 0.200-3.470 | 0.801 | 73/<10 | 0.571 | 0.268-1.215 | 0.146 | 73/<10 | 0.533 | 0.182-1.564 | 0.252 | 73/<10 | 0.696 | 0.315-1.538 | 0.370 |
| **Chemotherapy** |  |  |  |  |  |  |  |  |  |  |  |  |  |  |  |  |  |  |  |  |
| Anthracyclines | 2605/67 | 0.754 | 0.410-1.386 | 0.754 | 2605/95 | 1.729 | 0.983-3.042 | 0.058 | 2605/199 | 0.765 | 0.577-1.013 | 0.061 | 2605/186 | 0.778 | 0.610-0.994 | 0.044 | 2605/264 | 0.795 | 0.486-1.300 | 0.360 |
| Alkylating agents | 2878/84 | 0.837 | 0.484-1.448 | 0.525 | 2878/107 | 1.177 | 0.795-1.742 | 0.415 | 2878/232 | 1.031 | 0.725-1.467 | 0.866 | 2878/202 | 0.684 | 0.494-0.948 | 0.023 | 2878/313 | 1.397 | 0.849-2.299 | 0.188 |
| Platinum | 736/29 | 1.718 | 0.692-4.267 | 0.244 | 736/26 | 0.945 | 0.466-1.915 | 0.875 | 736/63 | 1.222 | 0.836-1.785 | 0.301 | 736/47 | 0.617 | 0.425-0.895 | 0.011 | 736/95 | 1.612 | 0.930-2.795 | 0.089 |
| Vinca-alkaloids | 4074/117 | 0.747 | 0.423-1.320 | 0.315 | 4074/155 | 1.462 | 0.876-2.438 | 0.146 | 4074/345 | 1.396 | 0.951-2.048 | 0.088 | 4074/304 | 0.981 | 0.617-1.560 | 0.936 | 4074/450 | 0.976 | 0.742-1.284 | 0.962 |
| Antimetabolites | 2618/75 | 1.691 | 0.910-3.142 | 0.096 | 2618/96 | 0.522 | 0.200-1.365 | 0.185 | 2618/166 | **0.497** | **0.349-0.709** | **<0.001** | 2618/207 | 1.128 | 0.763-1.669 | 0.546 | 2618/277 | 0.920 | 0.450-1.882 | 0.819 |
| Epipodophyllotoxins | 1180/48 | 1.268 | 0.640-2.511 | 0.496 | 1180/40 | 0.853 | 0.561-1.299 | 0.460 | 1180/97 | 1.195 | 0.869-1.643 | 0.273 | 1180/88 | 1.200 | 0.871-1.653 | 0.265 | 1180/130 | 0.786 | 0.482-1.282 | 0.334 |

Abbreviations: 95% CI: 95% confidence interval, CCS: Childhood Cancer Survivors, RHR: relative Hospitalization Ratio.
^1^ Groups are mutually exclusive

**Supplementary Table S6.** (continued)

|  | **V - Mental and behavioral disorders** | | | | **XIII - Diseases of the musculoskeletal system and connective tissue** | | | |
| --- | --- | --- | --- | --- | --- | --- | --- | --- |
|  | **n/n with event** | RHR | 95%CI | p-value | **n/n with event** | RHR | 95%CI | p-value |
| **Sex^1^** |  |  |  |  |  |  |  |  |
| Male | 3125/38 | Ref |  |  | 3125/291 | Ref |  |  |
| Female | 2482/35 | 0.848 | 0.498-1.444 | 0.544 | 2482/215 | 1.072 | 0.865-1.328 | 0.528 |
| **Age at diagnosis, years^1^** |  |  |  |  |  |  |  |  |
| 0-4 | 2543/31 | Ref |  |  | 2543/205 | Ref |  |  |
| 5-9 | 1519/26 | 1.855 | 0.999-3.445 | 0.050 | 1519/144 | 1.227 | 0.961-1.568 | 0.101 |
| 10-14 | 1193/11 | 0.790 | 0.376-1.659 | 0.534 | 1193/115 | 1.192 | 0.903-1.574 | 0.215 |
| 15-17 | 352/<10 | 1.281 | 0.440-3.732 | 0.650 | 352/42 | **1.967** | **1.198-3.230** | **0.007** |
| **Follow-up time** |  | 1.059 | 1.001-1.121 | 0.045 |  | **1.095** | **1.072-1.119** | **<0.001** |
| **Surgery** | 3797/46 | 0.585 | 0.259-1.321 | 0.197 | 3797/358 | 1.027 | 0.746-1.415 | 0.870 |
| **Radiotherapy** |  |  |  |  |  |  |  |  |
| Cranial RT | 1193/22 | 1.373 | 0.698-2.699 | 0.358 | 1193/107 | 0.929 | 0.686-1.257 | 0.633 |
| Spinal RT | 355/<10 | 1.573 | 0.380-6.514 | 0.532 | 355/25 | 1.035 | 0.557-1.923 | 0.913 |
| Total body irradiation | 200/<10 | 0.837 | 0.172-4.060 | 0.825 | 200/24 | 1.445 | 0.891-2.345 | 0.136 |
| RT Thorax | 351/<10 | 1.471 | 0.574-3.768 | 0.422 | 351/30 | 0.617 | 0.302-1.261 | 0.186 |
| Abdominalpelvic RT | 420/<10 | 1.253 | 0.491-3.201 | 0.637 | 420/43 | 1.346 | 0.799-2.266 | 0.264 |
| Neck RT | 218/<10 | 0.231 | 0.031-1.709 | 0.151 | 218/22 | 1.195 | 0.564-2.532 | 0.641 |
| RT Upper extremities | 41/0 | NA |  |  | 41/<10 | 2.352 | 0.943-5.866 | 0.067 |
| RT Lower extremities | 73/<10 | 0.867 | 0.128-5873 | 0.884 | 73/18 | **3.352** | **1.878-5.982** | **<0.001** |
| **Chemotherapy** |  |  |  |  |  |  |  |  |
| Anthracyclines | 2605/32 | 1.349 | 0.616-2.956 | 0.455 | 2605/228 | 1.118 | 0.867-1.441 | 0.389 |
| Alkylating agents | 2878/34 | 0.602 | 0.283-1.279 | 0.187 | 2878/257 | 0.962 | 0.743-1.247 | 0.772 |
| Platinum | 736/10 | 1.165 | 0.506-2.680 | 0.720 | 736/64 | 0.956 | 0.664-1.378 | 0.810 |
| Vinca-alkaloids | 4074/49 | 0.801 | 0.399-1.607 | 0.532 | 4074/355 | 0.875 | 0.673-1.139 | 0.322 |
| Antimetabolites | 2618/34 | 0.795 | 0.386-1.637 | 0.534 | 2618/213 | 0.877 | 0.591-1.301 | 0.513 |
| Epipodophyllotoxins | 1180/19 | 1.407 | 0.696-2.844 | 0.342 | 1180/100 | 1.069 | 0.776-1.474 | 0.682 |

Abbreviations: 95% CI: 95% confidence interval, CCS: Childhood Cancer Survivors, RHR: relative Hospitalization Ratio.
^1^ Groups are mutually exclusive
