# Supplementary material for: A detailed insight in the high risks of hospitalizations in long-term childhood cancer survivors—A Dutch LATER linkage study
Source: PLoS One. 2020 May 19;15(5):e0232708. doi: 10.1371/journal.pone.0232708 (PMC7236987; doi:10.1371/journal.pone.0232708)
Supplement: S8 Table — * Other tumors comprise (frequency tables are displayed in S1 Table): Germ cell tumors, trophoblastic tumors, and neoplasms of gonads (Gonadal carcinomas, Malignant gonadal germ cell tumors, Malignant extracranial and extragonadal germ cell tumors, Intracranial and intraspinal germ cell tumors, Other and unspecified malignant gonadal tumors)Other malignant epithelial neoplasms and malignant melanomas (Other and unspecified carcinomas, Skin carcinomas, Malignant melanomas, Nasopharyngeal carcinomas, Thyroid carcinomas, Adrenocortical carcinomas)Langerhans cell histiocytosisHepatic tumors (Hepatic carcinomas, Hepatoblastoma)RetinoblastomaOther and unspecified malignant neoplasms (DOCX) [file pone.0232708.s009.docx]

**Supplementary Table 8**. Multivariable risk factor analyses for the effect of primary cancer type on the number of hospitalizations among childhood cancer survivors.

|  |  | **RHR** | **95% CI** | **p-value** |
| --- | --- | --- | --- | --- |
| **Age at diagnosis** | 0-4 years | Ref |  |  |
|  | 5-9 years | 0.832 | 0.627-1.104 | 0.203 |
|  | 10-14 years | 0.630 | 0.479-0.829 | 0.001 |
|  | >14 years | 0.564 | 0.402-0.792 | 0.001 |
| **Follow-up time** | |  |  |  |
| **Sex** | Male | Ref |  |  |
|  | Female | 1.206 | 0.950-1.522 | 0.124 |
| **Tumor** | Leukemia | Ref |  |  |
|  | Hodgkin lymphoma | 1.116 | 0.774-1.609 | 0.557 |
|  | Non-Hodgkin lymphoma | 0.871 | 0.611-1.240 | 0.443 |
|  | Central nervous system tumor | **2.946** | **2.100-4.133** | **0.000** |
|  | Bone tumor | 1.315 | 0.898-1.925 | 0.160 |
|  | Soft tissue sarcoma | **1.958** | **1.112-3.447** | **0.020** |
|  | Renal tumor | 1.086 | 0.629-1.875 | 0.766 |
|  | Neuroblastoma | 0.899 | 2.100-4.133 | 0.643 |
|  | Other* | **2.233** | **1.512-3.298** | **0.000** |

* Other tumors comprise (frequency tables are displayed in Supplementary Table S1):

- Germ cell tumors, trophoblastic tumors, and neoplasms of gonads (Gonadal carcinomas, Malignant gonadal germ cell tumors, Malignant extracranial and extragonadal germ cell tumors, Intracranial and intraspinal germ cell tumors, Other and unspecified malignant gonadal tumors)
- Other malignant epithelial neoplasms and malignant melanomas (Other and unspecified carcinomas, Skin carcinomas, Malignant melanomas, Nasopharyngeal carcinomas, Thyroid carcinomas, Adrenocortical carcinomas)
- Langerhans cell histiocytosis
- Hepatic tumors (Hepatic carcinomas, Hepatoblastoma)
- Retinoblastoma
- Other and unspecified malignant neoplasms
