# Supplementary material for: A detailed insight in the high risks of hospitalizations in long-term childhood cancer survivors—A Dutch LATER linkage study
Source: PLoS One. 2020 May 19;15(5):e0232708. doi: 10.1371/journal.pone.0232708 (PMC7236987; doi:10.1371/journal.pone.0232708)
Supplement: S9 Table — (DOCX) [file pone.0232708.s010.docx]

**Supplementary Table S9.** Clinical characteristics in childhood cancer survivors by attained age groups

|  | **attained age <20** | | | **attained age 20-30** | | | **attained age 30-40** | | | **attained age 40-50** | | | **attained age >50** | | |  |
| --- | --- | --- | --- | --- | --- | --- | --- | --- | --- | --- | --- | --- | --- | --- | --- | --- |
| **Patient characteristics** |  |  |  | |  |  | |  |  | |  |  | |  | p-value | |
| *Sex - n (%)* |  |  |  | |  |  | |  |  | |  |  | |  | 0.117 | |
| Male | 413 | 54.9% | 1102 | | 58.0% | 983 | | 55.3% | 535 | | 54.0% | 119 | | 51.3% |  | |
| Female | 339 | 45.1% | 797 | | 42.0% | 794 | | 44.7% | 455 | | 46.0% | 113 | | 48.7% |  | |
| *Year of birth - n (%)* |  |  |  | |  |  | |  |  | |  |  | |  | <0.001 | |
| <1970 | 0 | 0.0% |  | | 0.0% | 43 | | 2.4% | 308 | | 31.1% | 232 | | 100.0% |  | |
| 1970-1985 | 90 | 12.0% | 242 | | 12.7% | 1734 | | 97.6% | 682 | | 68.9% | 0 | | 0.0% |  | |
| >1985 | 662 | 88.0% | 1651 | | 86.9% | 0 | | 0.0% | 0 | | 0.0% | 0 | | 0.0% |  | |
| **Tumor and treatment characteristics** |  |  |  | |  |  | |  |  | |  |  | |  |  | |
| *Age at diagnosis (in years) - n (%)* |  |  |  | |  |  | |  |  | |  |  | |  | <0.001 | |
| 0-4 | 593 | 78.9% | 960 | | 50.6% | 698 | | 39.3% | 286 | | 28.9% | 20 | | 8.6% |  | |
| 5-9 | 118 | 15.7% | 601 | | 31.6% | 489 | | 27.5% | 283 | | 28.6% | 40 | | 17.2% |  | |
| 10-14 | 41 | 5.5% | 295 | | 15.5% | 448 | | 25.2% | 309 | | 31.2% | 110 | | 47.4% |  | |
| 15-17 | 0 | 0.0% | 43 | | 2.3% | 142 | | 8.0% | 112 | | 11.3% | 62 | | 26.7% |  | |
| *Year of diagnosis - n (%)* |  |  |  | |  |  | |  |  | |  |  | |  | <0.001 | |
| ≤1974 | 0 | 0.0% |  | | 0.0% | 21 | | 1.2% | 176 | | 17.8% | 140 | | 60.3% |  | |
| 1975-1984 | 16 | 2.1% | 69 | | 3.6% | 554 | | 31.2% | 622 | | 62.8% | 92 | | 39.7% |  | |
| 1985-1994 | 156 | 20.7% | 756 | | 39.8% | 951 | | 53.5% | 192 | | 19.4% | 0 | | 0.0% |  | |
| ≥1995 | 580 | 77.1% | 1068 | | 56.2% | 251 | | 14.1% | 0 | | 0.0% | 0 | | 0.0% |  | |
| *Primary childhood cancer - n (%)* |  |  |  | |  |  | |  |  | |  |  | |  | <0.001 | |
| Leukemia | 307 | 40.8% | 681 | | 35.9% | 589 | | 33.1% | 297 | | 30.0% | 26 | | 11.2% |  | |
| Hodgkin lymphoma | 11 | 1.5% | 91 | | 4.8% | 155 | | 8.7% | 109 | | 11.0% | 17 | | 7.3% |  | |
| Non-Hodgkin lymphoma | 29 | 3.9% | 179 | | 9.4% | 200 | | 11.3% | 113 | | 11.4% | 22 | | 9.5% |  | |
| Central nervous system tumors | 136 | 18.1% | 267 | | 14.1% | 213 | | 12.0% | 99 | | 10.0% | 29 | | 12.5% |  | |
| Bone tumors | 18 | 2.4% | 80 | | 4.2% | 99 | | 5.6% | 87 | | 8.8% | 48 | | 20.7% |  | |
| Soft tissue sarcomas | 44 | 5.9% | 131 | | 6.9% | 121 | | 6.8% | 74 | | 7.5% | 36 | | 15.5% |  | |
| Renal tumors | 66 | 8.8% | 219 | | 11.5% | 171 | | 9.6% | 97 | | 9.8% | 14 | | 6.0% |  | |
| Neuroblastoma | 77 | 10.2% | 88 | | 4.6% | 98 | | 5.5% | 37 | | 3.7% |  | | 0.0% |  | |
| Other | 64 | 8.5% | 163 | | 8.6% | 131 | | 7.4% | 77 | | 7.8% | 37 | | 15.9% |  | |
| *Treatment modality - n (%)* |  |  |  | |  |  | |  |  | |  |  | |  | <0.001 | |
| Surgery only | 80 | 10.6% | 223 | | 11.7% | 173 | | 9.7% | 67 | | 6.8% | 25 | | 10.8% |  | |
| Chemotherapy ± surgery | 442 | 58.8% | 1179 | | 62.1% | 925 | | 52.1% | 261 | | 26.4% | 32 | | 13.8% |  | |
| Radiotherapy ± surgery | 35 | 4.7% | 91 | | 4.8% | 113 | | 6.4% | 107 | | 10.8% | 86 | | 37.1% |  | |
| Chemotherapy + Radiotherapy ± surgery | 182 | 24.2% | 395 | | 20.8% | 554 | | 31.2% | 548 | | 55.4% | 86 | | 37.1% |  | |
| No therapy/therapy unknown | 13 | 1.7% | 11 | | 0.6% | 12 | | 0.7% |  | |  |  | |  |  | |
| *Chemotherapy - n(%)* |  |  |  | |  |  | |  |  | |  |  | |  |  | |
| Anthracyclines | 334 | 44.4% | 100 | | 5.3% | 884 | | 49.7% | 347 | | 35.1% | 40 | | 17.2% | <0.001 | |
| Alkylating agents | 361 | 48.0% | 1045 | | 55.0% | 914 | | 51.4% | 485 | | 49.0% | 73 | | 31.5% | <0.001 | |
| Platinum agents | 194 | 25.8% | 314 | | 16.5% | 181 | | 10.2% | 47 | | 4.7% | 0 | | 0.0% | <0.001 | |
| Vinca alkaloids | 509 | 67.7% | 1412 | | 74.4% | 1323 | | 74.5% | 735 | | 74.2% | 95 | | 40.9% | <0.001 | |
| Antimetabolites | 351 | 46.7% | 914 | | 48.1% | 844 | | 47.5% | 458 | | 46.3% | 51 | | 22.0% | <0.001 | |
| Epipodophyllotoxins | 275 | 36.6% | 482 | | 25.4% | 349 | | 19.6% | 72 | | 7.3% |  | | 0.0% | <0.001 | |
| *Radiotherapy - n (%)* |  |  |  | |  |  | |  |  | |  |  | |  |  | |
| Head | 102 | 13.6% | 233 | | 12.3% | 389 | | 21.9% | 393 | | 39.7% | 76 | | 32.8% | <0.001 | |
| Neck |  | 0.0% | 38 | | 2.0% | 77 | | 4.3% | 73 | | 7.4% | 23 | | 9.9% | <0.001 | |
| Spinal | 43 | 5.7% | 95 | | 5.0% | 130 | | 7.3% | 80 | | 8.1% |  | | 0.0% | 0.001 | |
| Thorax | 17 | 2.3% | 65 | | 3.4% | 104 | | 5.9% | 127 | | 12.8% | 38 | | 16.4% | <0.001 | |
| Abdominopelvic | 30 | 4.0% | 102 | | 5.4% | 103 | | 5.8% | 149 | | 15.1% | 36 | | 15.5% | <0.001 | |
| Upper extremties |  | 0.0% |  | | 0.0% |  | | 0.0% | 12 | | 1.2% | 13 | | 5.6% | <0.001 | |
| Lower extremities |  | 0.0% | 12 | | 0.6% |  | | 0.0% | 21 | | 2.1% |  | | 0.0% | <0.001 | |
| Total body irradiaton | 45 | 6.0% | 64 | | 3.4% | 71 | | 4.0% | 20 | | 2.0% | 0 | | 0.0% | <0.001 | |
| *Other therapies - n (%)* |  |  |  | |  |  | |  |  | |  |  | |  |  | |
| Stem cell transplantation | 60 | 8.0% | 78 | | 4.1% | 59 | | 3.3% | 16 | | 1.6% | 0 | | 0.0% | <0.001 | |
| **Follow-up** |  |  |  | |  |  | |  |  | |  |  | |  |  | |
| Hospitalization y/n | 366 | 48.7% | 1136 | | 59.8% | 1243 | | 69.9% | 754 | | 76.2% | 183 | | 78.9% | <0.001 | |
